# Clinical and Immune Effects of Lenalidomide in Combination with Gemcitabine in Patients with Advanced Pancreatic Cancer

**DOI:** 10.1371/journal.pone.0169736

**Published:** 2017-01-18

**Authors:** Gustav J. Ullenhag, Fariba Mozaffari, Mats Broberg, Håkan Mellstedt, Maria Liljefors

**Affiliations:** 1 Department of Immunology, Genetics and Pathology, Uppsala University, Uppsala, Sweden; 2 Department of Oncology, Uppsala University Hospital, Entrance 78, Uppsala, Sweden; 3 Department of Oncology-Pathology, Cancer Center Karolinska (CCK), Karolinska Institutet, Stockholm, Sweden; 4 Department of Oncology, Danderyd University Hospital, Stockholm, Sweden; 5 Department of Oncology, Karolinska University Hospital Solna, Stockholm, Sweden; Catalan Institute of Oncology, SPAIN

## Abstract

**Purpose:**

To assess the immunomodulatory and clinical effects of lenalidomide with standard treatment of gemcitabine in patients with advanced pancreatic cancer.

**Patients and Methods:**

Patients with advanced pancreatic cancer were treated in first line with lenalidomide orally for 21 days of a 28 days cycle and the standard regimen for gemcitabine. In Part I, which we previously have reported, the dose of lenalidomide was defined (n = 12). In Part II, every other consecutive patient was treated with either lenalidomide (Group A, n = 11) or gemcitabine (Group B, n = 10) during cycle 1. From cycle 2 on, all Part II patients received the combination.

**Results:**

A significant decrease in the proliferative response of peripheral blood mononuclear cells and the frequency of DCs were noted in patients at baseline compared to healthy control donors while the frequencies of CD4+ and CD8+ T cells, NK-cells and MDSCs were significantly higher in patients compared to controls. In Group A, a significant increase in the absolute numbers of activated (HLA-DR+) CD4 and CD8 T cells and CD8 effector memory T cells (p<0.01) was noted during treatment. A statistical increment in the absolute numbers of Tregs were seen after cycle 1 (p<0.05). The addition of gemcitabine, reduced most lymphocyte subsets (p<0.05). In Group B, the proportion of lymphocytes remained unchanged during the study period. There was no difference in overall survival, progression free survival and survival rate at one year comparing the two groups.

**Discussion:**

Patients with advanced pancreatic carcinoma had impaired immune functions. Lenalidomide augmented T cell reactivities, which were abrogated by gemcitabine. However, addition of lenalidomide to gemcitabine seemed to have no therapeutic impact compared to gemcitabine alone in this non-randomized study.

**Trial Registration:**

ClinicalTrials.gov NCT01547260

## Introduction

Pancreatic cancer is characterised by aggressive growth and treatment resistance [1]. The majority of patients presents with advanced disease and the five-year survival rate is less than 5% [2]. Even those twenty percent of patients who are eligible for radical surgery including adjuvant chemotherapy have a poor prognosis with only 20% alive at 5 years [3].

For patients with advanced disease, gemcitabine is the standard treatment resulting in a median survival time of 5.7 months [4]. Combining gemcitabine and capecitabine improved overall survival (OS) but with a more pronounced toxicity profile compared to gemcitabine alone [5]. Triple chemotherapy (FOLFIRINOX) also increased OS compared to gemcitabine, but again with added toxicity [6]. Blocking the epidermal growth factor receptor (EGFR) with erlotinib in combination with gemcitabine significantly improved OS but only with two months and hand-foot side effects were common [7]. Overall survival was also extended by two months adding nabpaclitaxel to gemcitabine [8]. Regardless of currently available treatments regimens, survival of pancreatic cancer patients remains dismal and new therapies are warranted.

Lenalidomide (Revlimid®), is a thalidomide analogue that was initially approved by the U.S. Food and Drug Administration (FDA) and the European Medicine Agency (EMA) for multiple myeloma (MM) [9,10]. The compound may exert anti-tumor effects through anti-angiogenic activities [11] and by the expansion of tumor antigen-specific T cells, augmenting natural killer (NK)—cell cytotoxicity [12]. Furthermore, lenalidomide stimulates T-cells inducing proliferation, cytokine production, and cytotoxic activity [12–14] and inhibits TNF-α and interleukin 12 production [15,16].

Clinical effects of lenalidomide alone have been observed in patients with various advanced solid tumors [17–20] or in combination with chemotherapy [21]. Combined treatment with gemcitabine and lenalidomide of pancreatic carcinoma cells in vitro induced a higher tumor cell killing than with either agent alone [22]. Treatment of patients with metastatic pancreatic carcinoma using immunomodulatory drugs, such as pomalidomide or lenalidome in combination with gemcitabine, showed no clinical effects [23,24](23, 24). However, our study was initiated before the results from those studies were published.

Gemcitabine exerts a direct cytotoxic effect on tumor cells [23], but also augments immune responses contributing to a therapeutic effect [24]. Gemcitabine may activate T cells [25], increase the number of dendritic cells (DCs) [26], augment loading of antigens onto antigen-presenting cells (APC) [27], down-regulate the frequency of T-regulatory (Treg) cells [28], as well as myeloid derived suppressor cells (MDSC) [29] and increase the production of T cell derived IL-6 [30]. Administration of gemcitabine may also render tumor cells more susceptible to T-cell mediated destruction by up-regulation of death receptors [31]. Furthermore, gemcitabine has been shown to enhance immune responses against cancer vaccines [25]. The data support that lenalidomide and gemcitabine in combination may be of interest to explore for the therapy of pancreatic carcinoma.

We have conducted a study in chemo-naive patients with advanced pancreatic cancer combining lenalidomide and gemcitabine. Part I of the trial, defining the lenalidomide dose has previously been reported [32]. In part II, patients were first treated with lenalidomide or gemcitabine alone respectively,followed by the combination of these two agents. In this report, immune responses and clinical effects of part II are presented as well as survival data for all patients.

## Materials and Methods

**Fig 1:**
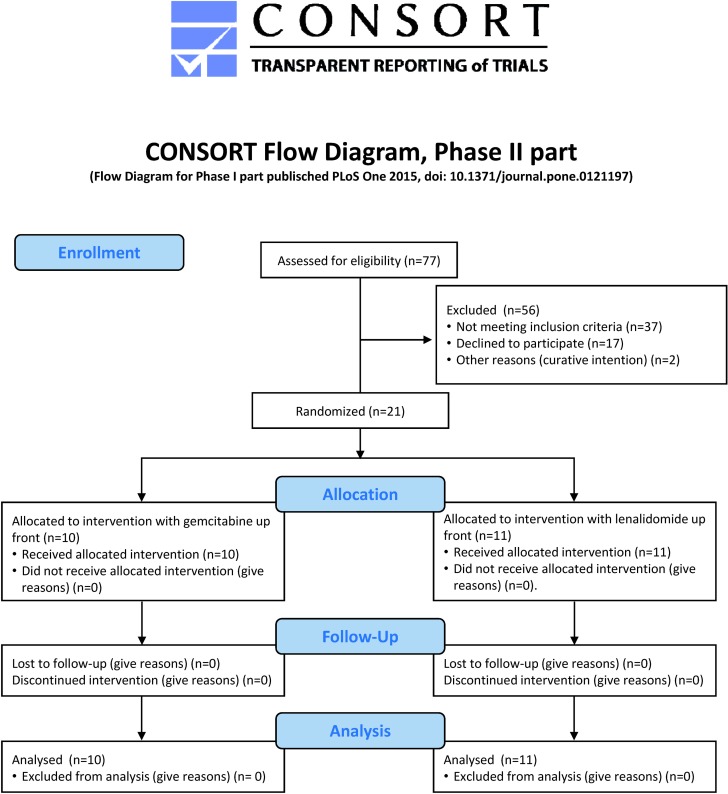
CONSORT Flow Diagram for the patients screened for and enrolled in part II. Corresponding data for patients enrolled in part I, has been published in Ullenhag GJ et al, PLOS ONE, 2015; 10(4).

The Protocol for this trial and supporting TREND Checklist are available as supporting information, see S1 File and S1 Fig. The CONSORT Flow Diagram is shown in Fig 1.

To our knowledge, all ongoing and related trials for this drug/intervention are registered.

The study (ClinicalTrials.gov identifier, NCT01547260) (https://register.clinicaltrials.gov/) was approved by the Regional Ethical Review Boards for Stockholm and Uppsala on the 6^th^ of October 2009 and by the Medical Products Agency Uppsala, Sweden. The study was registered at ClinicalTrials.gov only after inclusion began since registration was not a routine procedure in Sweden in 2009. Patients were treated according to the Declaration of Helsinki’s ethical principles for medical research involving human subjects. The trial was performed according to Good Clinical Practice guidelines. All patients provided an informed written consent prior to study entry.

### Patient population

Details regarding patients have been described elsewhere [32]. Briefly, eligible patients had histologically or cytologically confirmed unresectable, locally advanced, or metastatic adenocarcinoma of the pancreas. No prior chemotherapy for metastatic or locally advanced disease was allowed. Other eligibility criteria included: age >18 years, Eastern Cooperative Oncology Group (ECOG) performance status of 0 or 1, life expectancy > 12 weeks, adequate bone marrow, renal and hepatic functions as defined [32]. Patients enrolled in part I were recruited from the 14^th^ January 2010 to the 20^th^ May 2011, patients enrolled in part II were recruited from the 12^th^ October 2011 to the 13^th^ February 2013.All patients were followed up for survival every 3 month after discontinuation of the trial. The first follow up was on the 12^th^ October 2010 and the last follow up was on the 11^th^ November 2014.

### Study design and treatment schedule

This dual-agent, three-centre, open-label phase I/II study was conducted at the Karolinska University Hospital, Departments of Oncology, Solna (parts I and II) and Danderyd (part II), respectively, and Department of Oncology, Uppsala University Hospital, Uppsala, Sweden (parts I and II).

Thirteen patients were recruited to part I and 21 patients to part II. Patients, who received at least two cycles of the treatment according to the study protocol, were considered to be evaluable. If therapy was discontinued before completion of two treatment cycles for other reasons than AEs as per protocol, the subject was replaced.

The primary study objectives of part I was to determine MTD (Maximum Tolerated Dose) and safety. In part II, the primary objective was to evaluate immunomodulatory effects. For both parts of the study, the secondary objective was to evaluate clinical efficacy.

In part I, lenalidomide was administered orally once daily for 21 days of a 28 day cycle. The dose-escalation procedure of lenalidomide in part I has previously been described [32]. Gemcitabine was administered at a dose of 1000 mg/m^2^ intravenously for 30 minutes, at days 1, 8 and 15 every 28 days. MTD of lenalidomide in combination with the standard dose of gemcitabine was established to 25 mg/day days 1–21 of 28 [32]. In part II, every other consecutively included patient was treated with either lenalidomide (Group A) or gemcitabine (Group B) as monotherapy, during treatment cycle number 1. From treatment cycle number 2, all patients (part II) were treated with lenalidomide in combination with gemcitabine. This design was chosen since we wanted to evaluate the immunomodulatory effects of either drug separately, in addition to the combined treatment. The patients received prophylactic low molecular weight heparin (LMWH) (dalteparin, Pfizer Inc. New York, USA) (5000 IU s.c. once daily) during lenalidomide treatment.

### Patient evaluation

Patients were evaluated as previously described [32]. Progression-free survival (PFS) and OS were calculated from the time of start of study treatment until clinical and/or radiological signs of progression or until death, respectively. Survival rate at one year was calculated as the frequency of patients alive at week 52 after start of therapy. An electronic case report form (eCRF), PheedIt (SAS Institute) was used for recording.

### Immunoassays

In part II, peripheral blood samples were drawn for immune monitoring at three time-points; before treatment (baseline), at the end of treatment with lenalidomide or gemcitabine alone (after cycle 1) and after treatment with the combination of lenalidomide and gemcitabine (after cycle 2). Phenotyping of T cells (CD4, CD8), B cells and NK/NKT cells (CD16/CD56), Tregs and MDSCs was performed as described below.

### Monoclonal antibodies and other reagents

Antibodies conjugated with FITC, PE, PerCP, APC, AF700 and Pacific blue against the surface on intracellular molecules CD3, CD4, CD8, CD16, CD25, CD56, CD69, CD95, CD123, CD178, CD197, CD45RA, HLA-DR, CD11c, CD11b, CD127, Foxp3, Perforin, Granzyme B, IFN-γ, IL-17 as well as the anti-human lineage cocktail, CD3,CD14,CD19,CD20, CD56 including isotype-matched controls were purchased from Biolegend (Nordic Biosite, Täby, Sweden) and eBioscience, (San Diego, CA, USA). PMA and ionomycin were purchased from Sigma (St Louis, MO, USA) and Brefeldin A was obtained from BD (Mountain view, CA, USA).

### Cellular staining and flow cytometry

Peripheral blood mononuclear cells (PBMC) were isolated from heparinized blood by separation on a Ficoll–Isopaque gradient (Amersham Pharmacia Biotech AB, Uppsala, Sweden). PBMC were used for cytokines secretion (IL-17/IFNγ) assay in T cells (CD4+/CD8+), NK cells (CD3-CD56+) and NKT cells (CD3+CD56+) as well as for T cell proliferation assays.

Lysed blood was used for detecting Tregs (CD4+CD25+CD123-/low Foxp3+), effector/memory CD4+/CD8+ cells (CD45RA+/-/CCR7+/-), DCs (Lin-HLA-DR+CD16+CD11c+CD123+), MDSCs (Lin-HLA-DR-CD16-CD11b+CD33+), lymphocyte subsets and Perforin/Granzyme B secretions by T, NK and NKT cells. Briefly, 1x10^6^ cells per tube were incubated with the appropriate concentrations of antibodies or isotype controls for 30 min on ice. Intracellular staining of cells was done after surface staining, fixation, permeabilization, and incubation with specific antibodies. To assess the ability of T/NK/NKT cells to produce IL-17/IFNγ in response to stimuli, intracellular staining for cytokines was performed after treatment with PMA/Ionomycin for 3 hours. Treg staining was carried out according to the instruction of the manufacturer (eBioscience). Cells were analyzed using a LSRII (BD) and data analyzed by the Flowjo software (OR, USA) as described [33]. A positive staining was set at a fluorescence intensity displayed by<1% of the cells stained with the isotype control. Cell subsets are presented as absolute numbers (x 10^9^/L).

### Proliferation assay

PBMC were stimulated with PHA (10 μg/ml) (Gibco BRL, Grand Island, NY, USA) in a 96-well culture plate. Cultures were incubated for 5 days. 1 mCi / well 3H-thymidine (Amersham Pharmacia Biotech, Uppsala, Sweden) was added for the final 16–18 h. Incorporated radioactivity was measured in a β-counter (Micro β1450, Wallace, Turku, Finland). Results are presented as stimulation index (SI) and calculated as the ratio of radioactivity of cells incubated with PHA compared to control cultures [34].

### Healthy controls

Blood of healthy aged-match donors, recruited among health care and laboratory personnel, were used as controls.

### Statistical methods

Statistical analyses were done using StatView® (SAS Institute Inc. Version 5.0.1., USA) and Prismversion 6.0 (Graphpad software, Ca, USA). Frequency and intensity of adverse events (AE) and serious adverse events (SAE) are subjects to descriptive analysis. PFS and OS time curves were plotted using the Kaplan-Meier method. Differences between survival curves were tested using the log-rank statistics. The chi-square and Fisher´s exact tests were applied for comparison of distribution between groups. The one way ANOVA for repeated measures was used to calculate statistical significance for cell markers. The Mann-Whitney test was used for comparison of patients at baseline with healthy controls. There was no adjustment for multiplicity. Results were considered to be statistically significant for p<0.05.

## Results

### Patient characteristics

Thirteen patients were included in the part I study. The patients characteristics has been described elsewhere [32]. Twenty-one were included in the part II study (Group A: n = 11, Group B: n = 10). Clinical characteristics of the patients included in part II are shown in Table 1. Median time from diagnosis to start of treatment with lenalidomide and gemcitabine was 7 weeks (range 2–11 weeks).

**Table 1:** Patients baseline characteristics and number of patients per treatment arm in phase II.

Patient no.	Sex/age (years)	ECOG performance status	Site of metastasis at inclusion	Previoustreatment	Treatment Arm #	No. of treatment- cycles	No. of immune- samples	PFS (weeks)	OS (weeks)*
**201**	M/64	0	Peritoneum	None	A	4	3	10	25
**202**	F/68	1	Liver	None	B	2	3	8	32
**203**	F/61	0	LAPC w/o met*	Surgery (P)**	A	2	2	8	13
**204**	F/77	0	Liver	None	B	4	3	16	76
**205**	M/78	1	LAPC w/o met	None	A	1	1	NE ##	19
**206**	M/63	1	Multiple	None	B	1	2	NE	7
**207**	F/66	1	Peritoneum	Surgery (P)	A	5	3	19	84
**208**	F/58	0	LAPC w/o met	None	B	4	3	16	112
**209**	F/72	1	LAPC w/o met	None	A	6	3	23	79
**210**	F/69	0	Liver	None	B	9	3	42	48
**211**	F/73	0	Nodes	None	A	10	3	39	130
**212**	M/72	1	LAPC w/o met	None	B	4	3	17	85
**213**	M/51	0	Peritoneum	None	A	2	3	8	31
**214**	M/72	1	LAPC w/o met	None	B	2	3	8	27
**215**	M/62	1	Lungs	Surgery (C)	A	2	3	8	19
**216**	M/77	1	LAPC w/o met	None	B	2	3	8	17
**217**	M/77	1	Liver	None	A	1	1	NE	4
**218**	F/68	0	Liver	None	B	4	3	17	26
**219**	F/66	0	Liver	None	A	2	3	7	25
**220**	F/78	0	LAPC w/o met	None	B	15	3	66	74
**221**	F/63	1	Multiple	None	A	3	3	15	18

* LAPC w/o met = locally advanced pancreatic cancer without metastasis.

** Surgery (P) = surgery, palliative intention.

Surgery (C) = surgery, curative intention (pancreaticoduodenectomy).

# Arm A = lenalidomide monotherapy in cycle no. 1.

Arm B = gemcitabine monotherapy in cycle no. 1.

## NE = not evaluable.

### Treatment scheduling performance

Dose reductions and treatment delays for patients in part I has been described previously [32]. In part II, a total number of 85 cycles were initiated (12 with lenalidomide as monotherapy; 9 with gemcitabine as monotherapy and 64 with the combination). Dose-reductions were frequently seen both for lenalidomide and gemcitabine. In Group A, 44.7% (17/38) of the treatment cycles were dose-reduced. The corresponding figure for Group B was 42.5% (20/47).

In twenty-four cycles, reduction was due to haematologic toxicities (in 23 cycles due to leucopenia/neutropenia and in one cycle thrombocytopenia). In eleven cycles, reduction was associated with non-haematologic toxicities (in three cycles nausea/vomiting, in two cycles muscle/skeletal pain, in two cycles fatigue, in one cycle abdominal pain, in one cycle elevated ALAT/ASAT value, in one cycle vein thrombosis and in one cycle pulmonary embolism). In two patients (no 205 and 217) lenalidomide was permanently withdrawn at day 14 during cycle 1 (see below).

Treatment delays were uncommon. In four patients, four cycles were delayed due to nausea (n = 1) and viral infections (n = 3). In two patients, six cycles were delayed at the discretion of the patients. Median delay was 12 days (range 1–32 days).

Median number of treatment cycles was 2 (range 1–10) in Group A and 4 (range 1–15) in Group B. Median treatment duration time was 8 weeks (range 2–35 weeks) and 15 weeks (range 2–70 weeks) in Group A and B, respectively. For all patients, median treatment duration was 10 weeks (range 2–70 weeks).

### Immune responses

Peripheral blood was drawn at baseline in 19 of the 21 enrolled patients. All nineteen patients completed immune testing after cycle 1 (Group A, n = 9, Group B, n = 10) and 17 patients also after cycle 2 (Group A, n = 8, Group B, n = 9).

### Immune responses before treatment

**Fig 2:**
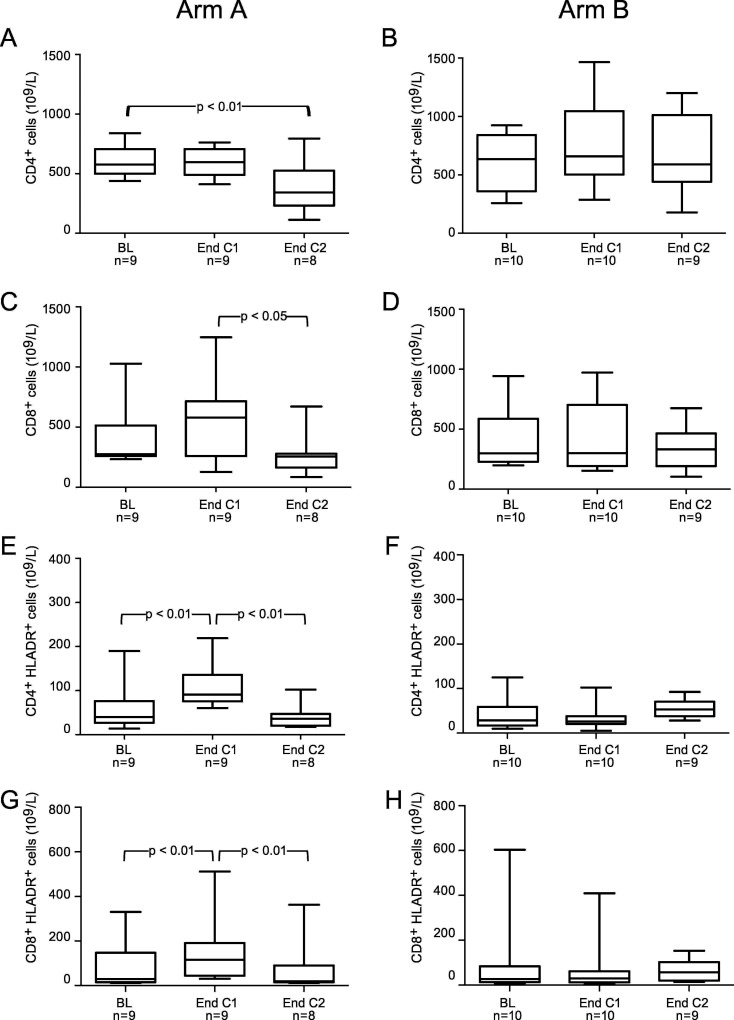
Absolute numbers of subsets of T cells at baseline (BL), at the end of cycle 1 (End C1) and at the end of cycle 2 (End C2) in part II patients treated with either lenalidomide monotherapy during cycle 1 with the addition of gemcitabine from cycle 2 (Arm A) (Left column) (Fig 2 A, C, E and G) or gemcitabine monotherapy during cycle 1 with the addition of lenalidomide from cycle 2 (Arm B) (Right column) (Fig 2 B, D, F and H). Changes in the absolute numbers of CD4+ T cells (A, B), CD8+ T cells (C, D), HLA-DR positive CD+4 T cells (E, F) and HLA-DR positive CD8+ T cells (G, H) over the treatment course, n = number of patients analysed at each time-point. *P*-values refer to the comparison with BL, End C1 and End C2 by one way ANOVA with repeated measures. The box, with a line indicating median, represents the 25^th^ and 75^th^ percentiles. The top and bottom whiskers represent the 90^th^ and 10^th^ percentiles, respectively.

**Fig 3:**
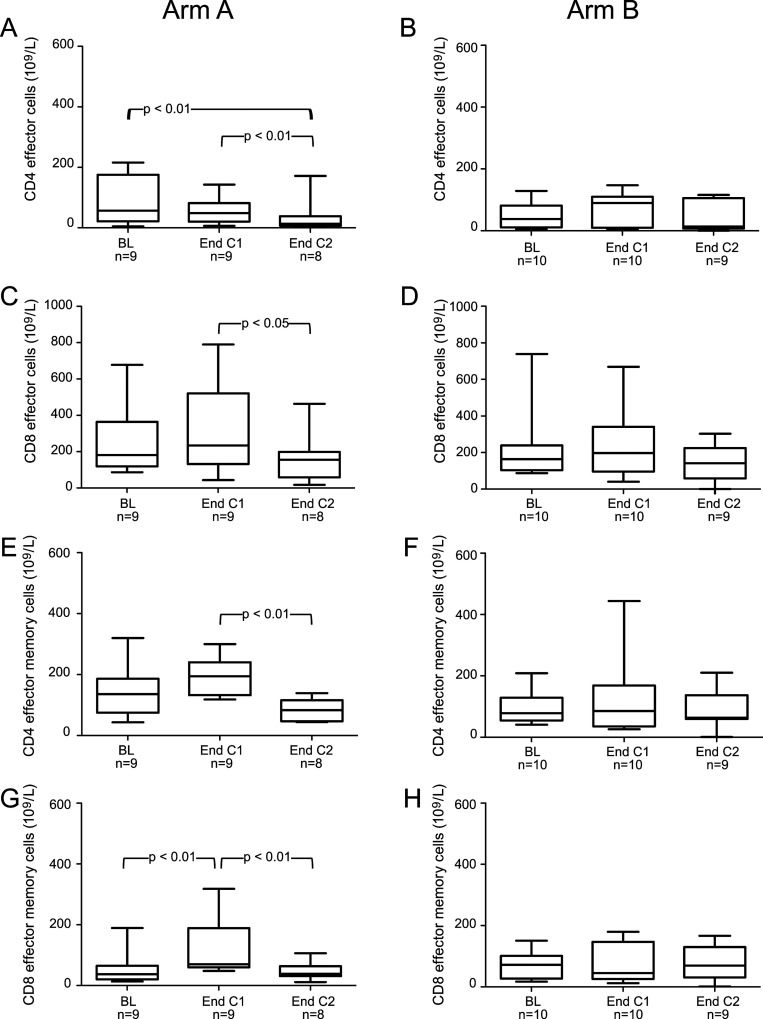
Absolute numbers of subsets of effector and effector memory T cells at baseline (BL), at the end of cycle 1 (End C1) and at the end of cycle 2 (End C2) in part II patients treated with either lenalidomide monotherapy during cycle 1 with the addition of gemcitabine from cycle 2 (Arm A) (Left column) (Fig 3 A, C, E and G) or gemcitabine monotherapy during cycle 1 with the addition of lenalidomide from cycle 2 (Arm B) (Right column) (Fig 3 B, D, F and H). Changes in the absolute numbers of CD8+ effector T cells (CD45RA+CCR7-CD8+) (C, D), CD8+ effector memory T cells (CD45RA-CCR7-CD8+) (G, H), CD4+ effector T cells (CD45RA+CCR7-CD4+) (A-B) and CD4+ effector memory T cells (CD45RA-CCR7-CD4+) (E, F) over the treatment course. n = number of patients analysed at each time-point. *P*-values refer to the comparison with BL, End C1 and End C2 by one way ANOVA with repeated measures. The box, with a line indicating median, represents the 25^th^ and 75^th^ percentiles. The top and bottom whiskers represent the 90^th^ and 10^th^ percentiles, respectively.

**Fig 4:**
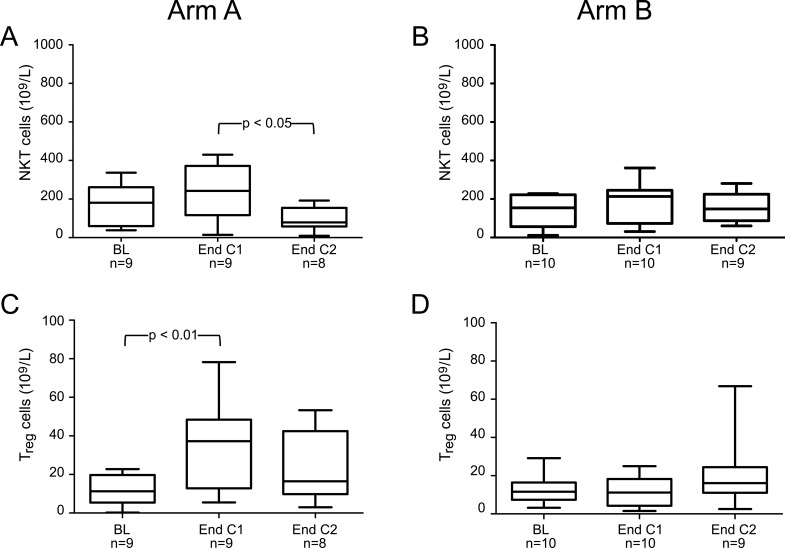
Absolute numbers of NKT-cells (CD3+CD56+CD16+) and regulatory T cells (T_reg_)(CD4+CD25+CD127-Foxp3+) at baseline (BL), at the end of cycle 1 (End C1) and at the end of cycle 2 (End C2) in part II patients treated with either lenalidomide monotherapy during cycle 1 with the addition of gemcitabine from cycle 2 (Arm A) (Left column) (Fig 4 A and C) or gemcitabine monotherapy during cycle 1 with the addition of lenalidomide from cycle 2 (Arm B) (Right column) (Fig 4 B and D). Changes in the absolute numbers of NKT-cells (A, B) and regulatory T cells (C, D) over the treatment course. n = number of patients analysed at each time-point. *P*-values refer to the comparison with BL, End C1 and End C2 by one way ANOVA with repeated measures. The box, with a line indicating median, represents the 25^th^ and 75^th^ percentiles. The top and bottom whiskers represent the 90^th^ and 10^th^ percentiles, respectively.

A significant decrease in T-cell proliferation (PHA-stimulation) and the frequency of DCs was noted in patients at baseline as compared to healthy control donors. However, the frequencies of CD4+, CD8+ and NK-cells, producing perforin and granzyme B, as well as MDSCs were significantly higher in patients compared to controls (Table 2). No difference in other immune tests comparing patients at baseline and healthy controls were noted (data not shown). There was no significant difference in immune responses at baseline comparing patients in Group A and B (Figs 2–4).

**Table 2:** Immune reactivity of patients with advanced pancreatic cancer before treatment compared to healthy donors.

Immune function	Patients Mean + SEM	(n)	Controls Mean + SEM	(n)	p value
PHA-stimulation of PBMC (SI)*	38.8 ± 13	(18)	102.7 ± 17.6	(19)	<0.01
**Lymphocyte subsets (%):**					
CD3+CD8+perforin	3.95 ± 1.1	(19)	0.7 ± 0.2	(11)	<0.01
CD3+CD4+perforin	2.2 ± 0.7	(19)	0.3 ± 0.05	(11)	<0.01
NK + perforin	12.3 ± 1.8	(19)	5.5 ± 1.0	(11)	<0.01
CD3+CD8+granzyme B	10.3 ± 2.0	(19)	5.2 ± 1.3	(11)	<0.05
CD3+CD4+granzyme B	3.0 ± 0.5	(19)	1.2 ± 0.3	(11)	<0.05
NK+granzyme B	12.0 ± 1.7	(19)	6.7 ± 0.6	(11)	<0.05
**Dendritic cells (%)**	0.08 ± 0.02	(19)	0.5 ± 0.1	(11)	<001
**Myeloid derived suppressor cells (%)**	0.33 ± 0.07	(19)	0.06 ± 0.01	(11)	<0.01

*Stimulation index.

### Immune responses during treatment

In Group A, a significant increase in the absolute numbers of activated CD4 and CD 8 T cells (HLA-DR+) (p < 0.01) was noted after cycle 1 (Fig 2E and 2G) but the addition of gemcitabine significantly reduced the total number of CD4 and CD8 T cells as well as the fraction of activated (HLA-DR+) T cells (Fig 2A, 2C, 2G and 2E).

CD8+ effector memory T cells (CD45RA-CCR7-CD8+) increased significantly after cycle 1 compared to baseline (p<0.01) (Fig 3G) and after adding gemcitabine CD4 and CD8 effector (Fig 3A and 3C) as well as effector memory T cells decreased significantly (Fig 3E and 3G).

A clear trend (p = 0.06) to an increase in absolute numbers of NK-T cells (CD3+CD16+CD56+) was observed after cycle 1 compared to baseline in Group A (Fig 4A) and after adding of gemcitabine, the numbers of NKT cells was reduced (p<0.05). No significant changes in NK cells were observed (data not shown). A statistically significant increment in the absolute numbers of regulatory T-cells (T_reg_) (CD4+CD25+CD127-FOXp3+) was also noted in Group A after cycle 1, compared to baseline (p<0.05) which was reduced to baseline after cycle 2 (Fig 4C).

In Group B, however, the proportion of absolute numbers of CD4+ and CD8+ T cells (Fig 2B and 2D), activated (HLR-DR^+^) CD4+ and CD8+ T cells (Fig 2F and 2H), CD4+ and CD8+ effector (Fig 3B and 3D) and effector memory T cells (Fig 3F and 3H), NK cells (data not shown), NK-T cells and Tregs (Fig 4B and 4D), did not change over time.

In both Group A and B, the frequencies of activated B cells (CD69+CD95+), CD4+ cells CD8 T cells and NK/NKT-cells producing perforin, granzyme B or IFNγ remained unchanged (data not shown)as well as the proportion of MDSCs and DCs (data not shown).

### Side-effects

Adverse events (AEs) and serious adverse events (SAEs) in part I have recently been described [32]. Hematological and non-hematological toxicities in part II are summarized in Table 3. Hematological AEs (all grades) were the most common, gastrointestinal (GI) intolerance and fatigue.

**Table 3:** Summary of maximum grade for toxicity in Ph II (Arm A and Arm B) (aggregate for all treatment cycles) (NCI CTCAE.V3.0).

Toxicity	Arm A (n = 11)	Arm B (n = 10)	Total (n = 21)	
	G* 1–4 No** (%)	G 1–4 No (%)	G 1–4 No (%)	G 3–4 No (%)
**Blood/bone marrow**				
Anemia	2(18)	3(30)	5(24)	0(0)
Leukopenia	7(64)	7(70)	14(67)	0(0)
Neutropenia	6(55)	6(60)	12(57)	6(28)
Thrombocytopenia	5(45)	6(60)	11(53)	0(0)
**Cardiac general**				
Hypotension	1(9)	0(0)	1(5)	0(0)
**Constitutional symptoms**				
Fatigue	11(100)	9(90)	20(95)	3(14)
Fever, in the absence of neutropenia (ANC <1.0 x 109/L)	2(18)	4(40)	6(28)	0(0)
Dysgeusia	2(18)	2(20)	4(19)	0(0)
**Dermatology/skin**				
Urticaria/Rash	1(9)	2(20)	3(14)	0(0)
Dry skin	0(0)	1(10)	1(5)	0(0)
Pruritus/itching	1(9)	3(30)	4(19)	0(0)
**Endocrine**				
Hypothyroidism	1(9)	0(0)	1(5)	0(0)
**Gastrointestinal**				
Constipation	2(18)	0(0)	2(9)	0(0)
Diarrhea	4(36)	5(50)	9(43)	0(0)
Dry mouth	2(18)	0(0)	2(9)	0(0)
Nausea	6(55)	5(50)	11(53)	0(0)
Vomiting	1(9)	2(20)	3(14)	1(5)
Anorexia	3(27)	4(40)	7(33)	1(5)
Stomatitis	0(0)	2(20)	2(9)	0(0)
Colonic stenosis	0(0)	1(10)	1(5)	1(5)
**Hepatobiliary/pancreas**				
Cholangitis	1(9)	0(0)	1(5)	1(5)
**Infection**				
Febrile neutropenia (ANC<1.0x109/L, fever>38.5°C)	1(9)	0(0)	1(5)	1(5)
Septicemia	0(0)	1(10)	1(5)	1(5)
Viral infection	0(0)	1(10)	1(5)	0(0)
**Lymphatics**				
Edema; limb	0(0)	3(30)	3(14)	0(0)
**Metabolic**				
ALAT elevated	4(36)	7(70)	11(53)	2(9)
ASAT elevated	3(27)	7(70)	10(47)	0(0)
**Musculoskeletal**				
Fracture	1(9)	0(0)	1(5)	1(5)
**Neurology**				
Dizziness	5(45)	1(10)	6(28)	2(9)
Neuropathy–sensory/motor	1(9)	3(30)	4(19)	0(0)
Somnolence	1(9)	0(0)	1(5)	1(5)
**Pain**				
Muscle	3(27)	1(10)	4(19)	0(0)
Abdominal	3(27)	1(10)	4(19)	2(9)
**Pulmonary/Upper respiratory**				
Dyspnea	1(9)	1(10)	2(9)	1(5)
Pneumonitis	0(0)	1(10)	1(5)	1(5)
**Secondary primary malignancy**	**0(0)**	**0(0)**	**0(0)**	**0(0)**
**Vascular**				
Thrombosis/thrombus/embolism	2(18)	1(10)	3(14)	2(9)

* = Grade.

** = Represents the number of subjects experiencing adverse events.

The incidence of neutropenia was 57% with 28% was of grade 3–4. There was one febrile neutropenia episode (5%) and one septicemia (5%). Thrombocytopenia and anemia (all grades) were noted in 53% and 24%, respectively, but only grade 1 or 2. No effects on lymphocyte, monocyte, eosinophil and basophil counts were noted (data not shown).

Fatigue was the most prominent non-hematological side-effect, noted in 95% of the patients, 14% was of grade 3. GI toxicities were common (diarrhea 53%, nausea 43% and anorexia 33%) and one episode of anorexia grade 3 as well as one of vomiting grade 3 were noted.

Elevation of ALAT or ASAT was reported in 53% and 47% (all grades), respectively, but only 9% were grade 3–4. There was one (5%) grade 2 hypothyroidism. No renal dysfunction was registered.

Dermatological toxicities (urticaria-rash/dry skin/pruritus-itching) were seen in 38%. Six patients (28%) had dizziness, 9% was of grade 3. Neuropathy, mainly neurosensory toxicity was noted in 19% (all grade 1–2). A grade 3 somnolence was seen in one patient.

There were three thromboembolic AEs, which occurred during or after cycle 2. Two were unilateral deep vein thrombosis (DVT) (one of grade 2 and one of grade 3). One patient had a grade 4 pulmonary embolism. Treatment with lenalidomide was hold during cycle 3 but restarted at a lower dose from cycle 4.

Twelve SAEs were reported. Five were classified as probably not related to the trial drugs but underlying disease: grade 3 abdominal pain in pats no 201 and 203; progressive disease (in pats no 220 and 217); and a grade 3 fracture of the femoral neck in pat no 205; constipation related to disease progression in pat 220 which required surgical intervention but the patient died 18 days postoperatively; in pat 217 requiring hospitalization and the patient died after 8 days due to rapid disease progression. One patient (pat no 218) required hospital admission at three occasions due to diarrhea (grade 3), dyspnoea (grade 3) and septicemia (grade 4), respectively. One patient was treated for febrile neutropenia (grade 3) (no 209) and another for cholangitis (grade 3) (no 207). Two SAEs were grade 3 vomiting (no 212) and grade 3 DVT (no 219) respectively.

### Clinical effects

All patients were evaluable for OS and survival rate at week 52. Twenty-nine patients completed at least two cycles of therapy and evaluable for PFS. In part I, one out of the twelve patients was withdrawn during cycle 1 due to vomiting related to underlying disease [32]. In part II, three out of the 21 patients were withdrawn before completion of two cycles: in two due serious adverse events (SAEs) (no 205 and 217, see above) and in another due to reduced performance status related to the underlying disease (pat no 206).

**Fig 5:**
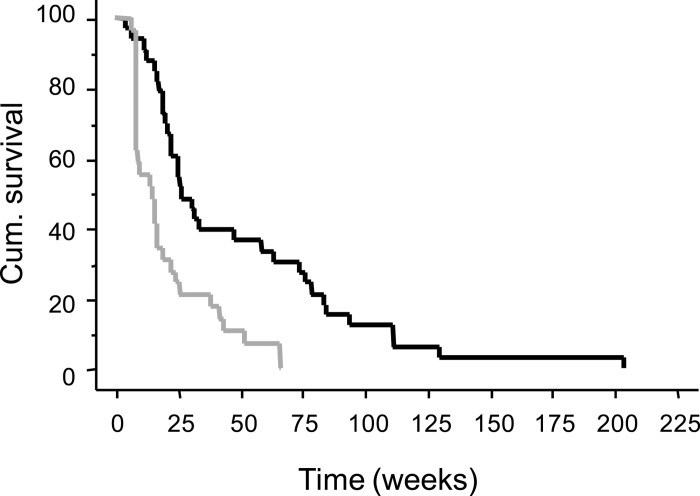
Cumulative overall survival (OS) (solid black line) and progression-free survival (PFS) (solid grey line) from the start of study treatment until death (OS) and clinical and/or radiological signs of disease progression (PFS), respectively, for all patients in the study. Number of evaluable patients for OS = 33 out of 33, for PFS = 29 out of 33.

Median PFS in part I was 14 weeks (range; 8–66), in part II, Group A 10 weeks (7–39), and in Group B 16 weeks (range; 8–66) and in all patients together 15 weeks (range; 7–66). Median OS time in part I was 28.5 weeks (range; 12–204); in part II, Group A 25 weeks (range; 4–130), in part II Group B 40 weeks (range; 7–112) and in all patients together 27 weeks (range; 4–204). The OS and PFS curves for all patients in the study are shown in Fig 5. The survival rate at one year was 42% in part I, 27% in Group A (part II), 40% in Group B (part II) and 36% in all patients together. There was no statistical difference in OS, PFS or survival rate at one year comparing patients within the different groups or between patients in part I and part II.

## Discussion

In this investigator-initiated phase I/II study, we examined the safety and therapeutic efficacy of a novel protocol using the immunomodulatory agent lenalidomide in combination with gemcitabine as first-line treatment of patients with advanced pancreatic cancer. The feasibility and tolerability of the combination regime in part I of the study, has recently been described [32].

In this report, the clinical effects for both part I and II are reported. This is the first study examining the immunomodulatory effects of lenalidomide as a single agent in patients with pancreatic cancer as compared to gemcitabine alone. Patients were shown to have impaired immune functions before entering the study. A significantly lower frequency of DCs and a significantly higher frequency of MDSCs compared to healthy controls were observed. These findings are in agreement with previous studies in patients with pancreatic cancer [35,36]. However, no difference in the total number of Tregs in patients compared to healthy volunteers could be noted in the present study while a significantly lower frequency was seen in patients compared to controls in a previous study [37]. Moreover, a significantly higher frequency of T-cells (CD4+, CD8+) and NK-cells producing perforin and granzyme B compared with healthy controls were noted, which might be related to the presence of tumor antigen-specific T cells as reported in pancreatic cancer patients [38]. Furthermore, the T cell proliferative capacity in response to mitogen was impaired, which is a common finding in cancer patients [39]. This might be due to increased immune suppression by MDSCs [40] and/or Tregs [41] as well as intrinsic T cell dysfunctions associated with eg. aberrant T cell signaling [42].

In cancer patients, lenalidomide treatment has been shown to augment the number of activated T cells [43] which was also noted in the present study. However, gemcitabine abrogated this effect. Our results are in contrast to a previous study showing that lenalidomide and gemcitabine in combination had clear immune-stimulatory effects [22]. The reasons for the contradictory results might be due to differences between artificial cancer cell lines and pancreatic cancer patients on treatment.

In preclinical and clinical studies, both gemcitabine [28,35] and lenalidomide [44,45] have been shown to inhibit the expansion and functional activity of Tregs.

However, in another study an increased number was observed with lenalidomide treatment CLL patients (Palma M, personal communication) which was in line with the results of Group A. Furthermore, although the numbers of Tregs decreased after adding gemcitabine to lenalidomide treatment, there was no reduction in Tregs of gemcitabine monotherapy (Arm B).

Also the results of lenalidomide on NK cells are conflicting. A small but not significant increase of NK cells in lenalidomide treated patients was observed in the present study while others have noted an increase in NK cells as well as NK cell mediated cytotoxicity and still others observed no effects on NK cell functions [46].

The median number of treatment cycles in both part I and II [32] was similar to that of Infante et al [47]. Median PFS in the present study was comparable to that of gemcitabine alone [4] and the combination with lenalidomide [47], while overall survival seemed to be superior in our study. The good performance status (ECOG 0–1) in our study and different second line treatments might explain the difference.

Side effects were mainly the same as to previous studies using lenalidomide or pomalidomide in combination with gemcitabine for advanced pancreatic cancer [47,48]. The overall side-effect profile was not more pronounced than expected. The frequency of grade 3–4 VTEs in this present trial was however lower than that noted when using aspirin as prophylactic anticoagulant [47]. No added toxicity was seen of the prophylactic LMWH schedule, as in concert with Maraveyas A et al [49].

In summary, patients with advanced pancreatic carcinoma had a suppressed immune system with reduced expansion of T and NK cells with a lytic capability. Lenalidomide seemed to expand activated T cells while addition of gemcitabine hampered those functions. Further studies are needed to explore the utility of immune-modulating agents in combination with gemcitabine in pancreatic carcinoma. Lenalidomide in combination with gemcitabine does not seem to be a rewarding treatment strategy. Other immunomodulatory approaches should be explored. Immune checkpoint antibodies might be an option to increase the therapeutic efficacy of gemcitabine.

## Supporting Information

S1 Fig TREND Checklist. (PDF)

S1 File Study protocol. (PDF)

## Abbreviations

OS: overall survival
PFS: progression-free survival
NK: natural killer–cell
DC: dendritic cell
Treg: T-regulatory cell
MDSC: myeloid derived suppressor cell
DVT: deep vein thrombosis
LMWH: low molecular weight heparin
PBMC: peripheral blood mononuclear cell
AE: adverse event
SAE: serious adverse event
