# Supplementary material for: Clinical and Immune Effects of Lenalidomide in Combination with Gemcitabine in Patients with Advanced Pancreatic Cancer
Source: PLoS One. 2017 Jan 18;12(1):e0169736. doi: 10.1371/journal.pone.0169736 (PMC5242484; doi:10.1371/journal.pone.0169736)
Supplement: S1 File — (PDF) [file pone.0169736.s002.pdf]

Protocol Title: Phase I/II study of lenalidomide and gemcitabine as first-line treatment in patients with locally advanced or metastatic pancreatic cancer

**STUDY DRUG**	**Lenalidomide (Revlimid®)**
**PROTOCOL NUMBER:**	**LENAGEM-PANC**
**VERSION:**	**Final Version 1.1**
**DATE:**	**09 July 2009**
**INCLUDING AMENDMENT:**	**Version 1.0**
**DATE:**	**19 Oct 2010**
**EUDRACT NUMBER:**	**2009-011793-14**

Sponsor: Prof. Håkan Mellstedt MD, PhD.
Karolinska University Hospital/Institute
Department of Oncology
SE- 171 76 Stockholm, Sweden
Phone: +46 8 51774641, 51774308
Fax: + 46 8 318327
e-mail: hakan.mellstedt@karolinska.se

CONFIDENTIAL
The information contained in this document is regarded as confidential and, except to the extent necessary to obtain informed consent, may not be disclosed to another party unless law or regulations require such disclosure. Persons to whom the information is disclosed must be informed that the information is confidential and may not be further disclosed by them.

PRINCIPAL INVESTIGATOR SIGNATURE PAGE

Principal Investigator:

Signature of Investigator Date

Maria Liljefors

Printed Name of Investigator

By my signature, I agree to personally supervise the conduct of this study and to ensure its conduct in compliance with the protocol, informed consent, EC procedures, the Declaration of Helsinki, ICH Good Clinical Practices guidelines, and local regulations governing the conduct of clinical studies.

STUDY PERSONNEL

**Sponsor:**	Prof Håkan Mellstedt Dept of Oncology, Karolinska University Hospital, Stockholm, Sweden
**Study Location:**	Karolinska University Hospital, Stockholm, Sweden<br>Akademiska sjukhuset/University Hospital, Uppsala
**Principal Investigator:**	Maria Liljefors

Table of Contents

1 PROTOCOL SYNOPSIS 6

2 SCHEDULE OF STUDY ASSESSMENTS – STUDY FLOW CHART. TABLES A (8), B (9) AND C (10). 8

3 GLOSSARY OF ABBREVIATIONS 11

4 BACKGROUND AND RATIONALE 13

4.1 Introduction 13
4.2 Indications and Usage of lenalidomide 14
4.3 Adverse Events 14
4.4 Rationale for combining lenalidomide and gemcitabine 14

5 STUDY OBJECTIVES AND ENDPOINTS 16

5.1 Phase I Study Objectives 16
5.1.1 Primary study objective 16
5.2 Phase II Study Objectives 16
5.2.1 Primary study objective 16
5.2.2 Secondary study objective 16
5.3 Endpoints 16
5.3.1 Primary Endpoints 16
5.3.2 Secondary Endpoints (phase II) 16

6 INVESTIGATIONAL PLAN 17

6.1 Overall design 17
6.1.1 Phase I part 17
6.1.2 Phase II part 20
6.1.3 Venous thromboembolism prophylaxis 22
6.1.4 Procedures for enrolment of eligible patients and patient numbering 22
6.2 Investigational Drug 22
6.2.1 Lenalidomide 22
6.2.2 Gemcitabine 25
6.3 Screening and Eligibility 26
6.3.1 Inclusion Criteria 26
6.3.2 Exclusion criteria 28
6.4 Visit schedule and assessments 29
6.5 Drug Administration 29
6.5.1 Treatment assignments 29
6.5.2 Dosing regimen 30
6.5.3 Record of administration 30
6.6 Dose Continuation, Modification and Interruption 30
6.6.1 Instructions for dose continuation, modification and interruption 30
6.6.2 Dose reduction steps for Lenalidomide and gemcitabine 31
6.6.3 Instructions for dose modifications or interruption during a cycle 31
6.6.4 Dosing adjustment Guidelines for initiation of a new cycle of therapy 33
6.6.5 Treatment compliance 34
6.7 Concomitant therapy 34
6.7.1 Recommended concomitant therapy 34
6.7.2 Prohibited concomitant therapy 34
6.8 Discontinuation of Study Treatment 34
6.9 Follow-Up 35

7 ADVERSE EVENTS 36

7.1 Adverse Event 36

7.1.1 Abnormal laboratory values defined as adverse events *36*
7.2 Serious adverse event **36**
7.2.1 Pregnancies *37*
7.3 Classification of severity **38**
7.4 Classification of Relationship/Causality of adverse events (SAE/AE) to study drug **39**
7.5 Serious Adverse Event (SAE) Reporting **39**
7.5.1 Immediate reporting by Investigator to Sponsor and Sponsor reporting to Celgene *39*
7.5.2 Reporting to Regulatory Authorities and the Ethics Committee *40*
7.6 Adverse event updates **41**

8 ASSESSMENT OF STUDY OBJECTIVES 42

8.1 Assessment of safety **42**
8.2 Assessment of Immunomodulation **42**
8.3 Assessment of clinical efficacy **43**
8.3.1 Following parameters will be described, according to section 10: *43*
8.4 Protocol amendments **43**
8.5 Protocol deviations **44**

9 DATA MANAGEMENT 45

9.1 Analyses and Reporting **45**
9.2 Study monitoring and auditing **45**
9.2.1 Investigator responsibilities *45*

10 BIOSTATISTICAL ANALYSIS 46

10.1 Overview **46**
10.2 Datasets to be analyzed **46**
10.2.1 Primary endpoint (phase I) *46*
10.2.3 Secondary endpoint (phase II) *46*
10.3 Statistical Methodology **47**
10.4 Safety evaluation **47**
10.5 Sample size **47**

11 REGULATORY CONSIDERATIONS 48

11.1 Ethics Committee approval **48**
11.2 Informed consent **48**
11.3 Subject confidentiality **48**
11.4 Study records requirements **49**
11.5 Premature discontinuation of study **49**

12 REFERENCES 50

Appendix 1 – ECOG Performance Status Scale **52**
Appendix 2 – NCI CTCAE Version 3.0 ..... **53**
Appendix 3 – Immunological monitoring Test panel of Phase I/II study of lenalidomide and gemcitabine in advanced pancreatic cancer **54**
Appendix 4 – SAE Form **55**
Appendix 5 – Patientens Informerade Samtycke Phase I **1**
Appendix 6 – Patientens Informerade Samtycke Phase II **1**
Appendix 7 – World Medical Association Declaration of Helsinki **1**

1 Protocol Synopsis

**PROTOCOL TITLE:** Phase I/II study of gemcitabine and lenalidomide as first-line treatment in subjects with locally advanced or metastatic pancreatic cancer	
**PROTOCOL NUMBER:**	LENAGEM-PANC
**DATE PROTOCOL FINAL:**	
**STUDY DRUGS:**	Revlimid®, lenalidomide and Gemzar®, gemcitabine
**INDICATION:**	Locally advanced or metastatic pancreatic cancer
**STUDY PHASE:**	Phase I/II

BACKGROUND AND RATIONALE:

Pancreatic cancer is characterised by aggressive growth, treatment resistance and an extremely poor prognosis. In subjects with locally advanced or metastatic disease, the median survival is approximately 6 – 11 months and 2 – 6 months, respectively. The currently accepted treatment for this disease in EU is gemcitabine. A promising approach could be the pharmacological inhibition of tumour angiogenesis with anti-vascular endothelial growth factor (VEGF) agents. VEGF is known to promote growth of pancreatic cancers and high expression is an indicator of poor prognosis.

Furthermore, preclinical studies indicate that pancreatic cancer is suppressed by inhibitors of VEGF, and the importance of angiogenesis in pancreatic cancer implies that anti-angiogenics may also be important in the treatment. Lenalidomide belongs to a proprietary class of compounds called immunomodulatory drugs (IMiDs). IMiDs have both immunomodulatory and anti-angiogenic properties which could confer antitumour and antimetastatic effects. Lenalidomide has been demonstrated to possess anti-angiogenic activity through inhibition of bFGF, VEGF and TNF-alpha induced endothelial cell migration. The effect of IMiDs on innate cell function in subjects with solid tumours has not yet been investigated.

STUDY OBJECTIVES:

Primary:

Phase I: To determine the MTD (Maximum Tolerated Dose) and safety of the regimen lenalidomide and gemcitabine as first-line treatment in subjects with pancreatic cancer.

Phase II: To evaluate the immunomodulatory effects of the regimen lenalidomide and gemcitabine as first-line treatment in subjects with pancreatic cancer.

Secondary:

Phase II: To evaluate time to progression, survival rate at 12 months and overall survival of lenalidomide in combination with gemcitabine in subjects with advanced pancreatic cancer as first-line treatment.

STUDY DESIGN:

This is a phase I/II open-label, multi-center study. It will consist of a phase I dose-finding part and a phase II part during which subjects will be treated at the MTD established during phase I. Lenalidomid will be administered by a stepwise dose-escalation schedule in the phase I part.

STUDY ENDPOINTS

Primary Phase I:

- MTD and safety (type, frequency, and severity) and relationship of AEs to lenalidomide and gemcitabine treatment.

Primary Phase II:

- Immunomodulatory effects.

Secondary (Phase II):

- Safety (type, frequency, and severity) and efficacy.
- Progression-free survival.
- Overall survival.
- Survival rate at 12 months.

**STUDY DURATION:** Approximately 2,5 years	**TOTAL SAMPLE SIZE:** Maximally 30 subjects
**DOSING REGIMEN(S):**<br><br>Lenalidomide will be administered orally once daily for 21 days followed by 7 days rest. The MTD of lenalidomide will be defined as the highest dose level at which no more than 1 out of 4 subjects experiences a DLT when administered in combination with gemcitabine. 3 subjects will be enrolled into each cohort for 15 mg, 20 mg and 25 mg respectively. Gemcitabine 1000 mg/m2 in 0.9% sodium chloride will be administered as intravenous infusion over 30 minutes, weekly for 3 weeks then rest for 1 week (days 1, 8, 15 of 28).	**STUDY DRUG SUPPLIES:**<br><br>Celgene Corporation will supply Revlimid®, lenalidomide as 5, 10, 15, 20 and 25 mg capsules.<br><br>Gemzar®, gemcitabine will be supplied according to standars health care procedures, as it is commercially available.

2 Schedule of Study Assessments – Table A. Study flow chart. Phase I group

	Screening (≤ 21 days from Baseline (BL))	BL	Cycle 1						Cycle 2				Cycle 3 and beyond					
			Day 1 §	Day 4 or 5	Day 8	Day 11 or 12	Day 15	Day 21 or 22 or 23	Day 1	Day 8	Day 15	Day 21 or 22 or 23	Day 1	Day 8	Day 15	Day 21 or 22 or 23	End of treatment or discontinuation from study drug	Survival Follow-up
Week (w)		-1	1		2		3	4	5	6	7	8	9,13 etc	10,14 etc	11,15 etc	12,16 etc		
Informed consent **	X																	
Medical history	X																	
Physical examination [1]	X	x							X				X				X	
Performance status	X	x	X		X		X	X	X	X	X		X				X	
Electrocardiogram	X																X	
Concomitant medication	X	x							X				X				X	
Serum chemistry [2]	X								X***				X***				X	
CA19-9	X								X***				X***				X	
Hematology incl differential	X		X***	X	X***	X	X***	X	X***	X***	X***	X	X***	X***	X***		X	
CT of the abdomen and X-ray*	X											X					X	
Pregnancy test and counselling [4 5]	X		X						X				X				X	
Dispense of Cycle 1 study drug			X															
Dispense study drug for next cycle									X				X					
Perform drug accountability								X				X				X#	X	
Adverse events			X		X		X	X	X	X	X	X	X	X	X	X#	X	
Gemcitabine			X		X		X		X	X	X		X	X	X			
Lenalidomide			Days 1–21 of 28						Days 1-21 of 28				Days 1-21 of 28					
Survival status [8]																		X
Vital signs [3]	X	x	X		X		X	X	X			X	X					
Immunomonitoring [6]																		
Collection of blood sample [7]																		

1) Incl.height (only at pre-treatment), weight.
2) Serum Chemistry: Na, K, Ca, creatinine, ASAT, ALAT, LD, γGT, ALP, bilirubin, LD. To include APT and INR at pre-treatment and TSH at pre-treatment, end of cycle 2 and every second months thereafter.
3) Blood pressure, heart rate.
4) Pregnancy test for women of child-bearing potential only (urine).
5) Must occur within 24 hours prior to initiation of lenalidomide or in the 3 days prior to the initiation of lenalidomide then monthly while on therapy (days -3 to day 1) and 30 days post the last dose of lenalidomide. All patients must be counselled monthly about pregnancy precautions and risks of fetal exposure (see protocol).
6) Immunomonitoring within 28 days before start of treatment. 8 heparin tubes (approximately 70-80 ml) for research test are sent to CCK, R8:01, Karolinska University Hospital.
7) Separation of PBMC to be stored at -136°C.
8) Every 3 month after discontinuation of this trial. Follow-up of subjects could be performed via telephone. Contact to be documented in the medical file.

§ Up to 7 days from Baseline.
* Within 3 weeks before start of treatment, thereafter every 2nd month. CT scan of the abdomen and X-ray or CT scan of the chest.
** Within 4 weeks before start of treatment.
*** Blood tests could be performed day -1 to day 1, day 7-8 day 14-15.
\# AE-registration and Drug accountability may be performed at Day 28-visit before start of next cycle.

Table B. Study flow chart. Phase II group Lenalidomide start

	Screening (< 21 days from Baseline (BL))	BL	Cycle 1						Cycle 2				Cycle 3 and beyond				End of treatment or discontinuation from study drug	Survival Follow-up
			Day 1§	Day 4 or 5	Day 8	Day 11 or 12	Day 15	Day 21 or 22 or 23	Day 1	Day 8	Day 15	Day 21 or 22 or 23	Day 1	Day 8	Day 15	Day 21 or 22 or 23		
Week (w)		-1	1		2		3	4	5	6	7	8	9,13 etc	10,14 etc	11,15 etc	12,16 etc		
Informed consent **	X																	
Medical history	X																	
Physical examination [1]	X	x							X				X				X	
Performance status	X	x	X		X		X	X	X	X	X		X				X	
Electrocardiogram	X																X	
Concomitant medication	X	x							X				X				X	
Serum chemistry [2]	X								X***				X***				X	
CA19-9	X								X***				X***				X	
Hematology incl differential	X		X***		X***		X***	X	X***	X***	X***	X	X***	X***	X***		X	
CT of the abdomen and X-ray*	X											X					X	
Pregnancy test and counselling [4 5]	X		X						X				X				X	
Dispense of Cycle 1 study drug			X															
Dispense study drug for next cycle									X				X					
Perform drug accountability								X				X				X#	X	
Adverse events			X		X		X	X	X	X	X	X	X	X	X	X#	X	
Gemcitabine									X	X	X		X	X	X			
Lenalidomide			Days 1–21 of 28						Days 1-21 of 28				Days 1-21 of 28					
Survival status [8]																		X
Vital signs [3]	X	x	X		X		X	X	X			X	X					
Immunomonitoring [6]	X							X				X						
Collection of blood sample [7]	X							X				X						

1) Incl.height (only at pre-treatment), weight.
2) Serum Chemistry: Na, K, Ca, creatinine, ASAT, ALAT, LD, γGT, ALP, bilirubin, LD. To include APT and INR at pre-treatment and TSH at pre-treatment, end of cycle 2 and every second months thereafter.
3) Blood pressure, heart rate.
4) Pregnancy test for women of child-bearing potential only (urine).
5) Must occur within 24 hours prior to initiation of lenalidomide or in the 3 days prior to the initiation of lenalidomide then monthly while on therapy (days -3 to day 1) and 30 days post the last dose of lenalidomide. All patients must be counselled monthly about pregnancy precautions and risks of fetal exposure (see protocol).
6) Immunomonitoring within 28 days before start of treatment. 8 heparin tubes (approximately 70-80 ml) for research test are sent to CCK, R8:01, Karolinska University Hospital.
7) Separation of PBMC to be stored at -136°C.
8) Every 3 month after discontinuation of this trial. Follow-up of subjects could be performed via telephone. Contact to be documented in the medical file.

§ Up to 7 days from Baseline.
* Within 3 weeks before start of treatment, thereafter every 2nd month. CT scan of the abdomen and X-ray or CT scan of the chest.
** Within 4 weeks before start of treatment.
*** Blood tests could be performed day -1 to day 1, day 7-8 day 14-15.
\# AE-registration and Drug accountability may be performed at Day 28-visit before start of next cycle.

Table C. Study flow chart. Phase II group Gemcitabine start

	Screening (≤ 21 days from Baseline (BL))		Cycle 1						Cycle 2				Cycle 3 and beyond					
		BL	Day 1§	Day 4 or 5	Day 8	Day 11 or 12	Day 15	Day 21 or 22 or 23	Day 1	Day 8	Day 15	Day 21 or 22 or 23	Day 1	Day 8	Day 15	Day 21 or 22 or 23	End of treatment or discontinuation from study drug	Survival Follow-up
Week (w)		-1	1		2		3	4	5	6	7	8	9,13 etc	10,14 etc	11,15 etc	12,16 etc		
Informed consent **	X																	
Medical history	X																	
Physical examination [1]	X	x							X				X				X	
Performance status	X	x	X		X		X	X	X	X	X		X				X	
Electrocardiogram	X																X	
Concomitant medication	X	x							X				X				X	
Serum chemistry [2]	X								X***				X***				X	
CA19-9	X								X***				X***				X	
Hematology incl differential	X		X***		X***		X***	X	X***	X***	X***	X	X***	X***	X***		X	
CT of the abdomen and X-ray*	X											X					X	
Pregnancy test and counselling [4 5]	X		X						X				X				X	
Dispense of Cycle 1 study drug			X															
Dispense study drug for next cycle									X				X					
Perform drug accountability								X				X				X#	X	
Adverse events			X		X		X	X	X	X	X	X	X	X	X	X#	X	
Gemcitabine			X		X		X		X	X	X		X	X	X			
Lenalidomide									Days 1-21 of 28				Days 1-21 of 28					
Survival status [8]																		X
Vital signs [3]	X	x	X		X		X	X	X			X	X					
Immunomonitoring [6]	X							X				X						
Collection of blood sample [7]	X							X				X						

1) Incl.height (only at pre-treatment), weight.
2) Serum Chemistry: Na, K, Ca, creatinine, ASAT, ALAT, LD, γGT, ALP, bilirubin, LD. To include APT and INR at pre-treatment and TSH at pre-treatment, end of cycle 2 and every second months thereafter.
3) Blood pressure, heart rate.
4) Pregnancy test for women of child-bearing potential only (urine).
5) Must occur within 24 hours prior to initiation of lenalidomide or in the 3 days prior to the initiation of lenalidomide then monthly while on therapy (days -3 to day 1) and 30 days post the last dose of lenalidomide. All patients must be counselled monthly about pregnancy precautions and risks of fetal exposure (see protocol).
6) Immunomonitoring within 28 days before start of treatment. 8 heparin tubes (approximately 70-80 ml) for research test are sent to CCK, R8:01, Karolinska University Hospital.
7) Separation of PBMC to be stored at -136°C.
8) Every 3 month after discontinuation of this trial. Follow-up of subjects could be performed via telephone. Contact to be documented in the medical file.

§ Up to 7 days from Baseline.
* Within 3 weeks before start of treatment, thereafter every 2nd month. CT scan of the abdomen and X-ray or CT scan of the chest.
** Within 4 weeks before start of treatment.
*** Blood tests could be performed day -1 to day 1, day 7-8 day 14-15.
# AE-registration and Drug accountability may be performed at Day 28-visit before start of next cycle.

3 Glossary of Abbreviations

**AE**	Adverse event
**ALAT (SGPT)**	Alanine transaminase (serum glutamate pyruvic transaminase)
**ALC**	Absolute lymphocyte count
**ANC**	Absolute neutrophil count
**ASCO**	American Society of Clinical Oncology
**AST (SGOT)**	Asparate transaminase (serum glutamic oxaloacetic transaminase)
**bFGF**	basic Fibroblastic Growth Factor
**CBC**	Complete blood count
**CFR**	Code of Federal Regulations
**CNS**	Central nervous system
**CRF**	Case report form
**CT**	Computed tomography
**CTC**	Common toxicity criteria
**CVA**	Cerebrovascular Accident
**DLT**	Dose-limiting toxicity
**DMC**	Data Monitoring Committee
**DVT**	Deep Vein Thrombosis
**EC**	Ethics Committee
**ECG**	Electrocardiogram
**ECOG**	Eastern Cooperative Oncology Group
**EMEA**	European Agency for Evaluation of Medicinal Products
**FCBP**	Female of child bearing potential
**FDA**	Food and Drug Administration
**GCP**	Good Clinical Practice
**ICH**	International Conference on Harmonization
**IL**	Interleukin
**IMiD**	Immunomodulatory Drug
**IND**	Investigational New Drug
**IRB**	Institutional Review Board
**LDH**	Lactate dehydrogenase
**MDS**	Myelodysplastic syndrome
**MedDRA**	Medical Dictionary for Regulatory Activities

**MTD**	Maximum Tolerated Dose
**NCI**	National Cancer Institute
**NK**	Natural Killer Cells
**NSAID**	Non-steroidal anti-inflammatory drug
**OS**	Overall survival
**PD**	Progressive disease
**PE**	Pulmonary Enbolism
**PFS**	Progression free survival
**RBC**	Red blood cell
**SAE**	Serious Adverse Event
**SOP**	Standard Operating Procedure
**TNFα**	Tumor Necrosis Factor Alpha
T_{reg}	Regulatory T Cells
**TSH**	Thyroid stimulating hormone
**TTP**	Time to progression
**ULN**	Upper limit of normal
**VEGF**	Vascular endothelial growth factor
**WBC**	White blood cell count
**β-hCG**	Beta-human chorionic gonadotropin hormone

4 Background and Rationale

4.1 Introduction

Pancreatic cancer is characterised by aggressive growth, treatment resistance and an extremely poor prognosis (1). In subjects with locally advanced or metastatic disease, the median survival is approximately 6 – 11 months and 2 – 6 months, respectively. The currently accepted treatment for this disease in EU is gemcitabine which supplanted treatment with 5-FU after it was shown that median survival duration was marginally improved (4.41 and 5.65 months respectively, p = 0,022) (2). The reported median survival time for subjects treated with single-agent gemcitabine in randomized phase III studies ranged form 4.9 to 7.2 months (3, 4).

Despite these improvements in the treatment of pancreatic cancer, the prognosis remains very poor. Consequently many studies have been carried out using gemcitabine in combination with other chemotherapeutics. Among the best of these is gemcitabine plus capecitabine or gemcitabine plus erlotinib, for which randomized phase III trials have been published.

Cunningham et al demonstrated an improved median overall survival using gemcitabine in combination with capecitabine compared to gemcitabine alone (6.0 and 7.4 months respectively, p = 0,026) and an overall survival benefit (HR 0,8, p = 0,26) (5). This, however, proved to have greater toxicity than single agent therapies. Gemcitabine in combination with erlotinib, showed a significant improvement in overall survival (median OS = 6.24 months), versus gemcitabine alone (5.9 months, p=0.025) (7).

A promising approach could be the pharmacological inhibition of tumour angiogenesis with anti-vascular endothelial growth factor (VEGF) agents, such as bevacizumab, cyclooxygenase-2 inhibitors (celecoxib), thalidomide and others. VEGF signalling appears to play a role in the progression of the disease; VEGF is known to promote growth of pancreatic cancers and high expression is an indicator of poor prognosis. Furthermore, preclinical studies indicate that pancreatic cancer is suppressed by inhibitors of VEGF.

Kindler et al demonstrated (6), in a phase II clinical trial 52 advanced pancreatic cancer subjects, that bevacizumab plus gemcitabine resulted in a median overall survival of 8.8 months (marginally better than expected for gemcitabine alone). Furthermore, promising results were found when thalidomide was tested in combination with celecoxib and gemcitabine (8). Despite the lack of a radiographic response, this study demonstrated biochemical response (decreased in serum CA 19-9) to treatment in 5 /12 subjects and overall mean survival of 10 months.

The preclinical data indicating the importance of angiogenesis in pancreatic cancer imply that anti-angiogenics may be important in the treatment of pancreatic cancer. Lenalidomide belongs to a proprietary class of Celgene compounds called

immunomodulatory drugs (IMiDs). IMiDs, of which thalidomide is the parent compound, have both immunomodulatory and anti-angiogenic properties which could confer antitumour and antimetastatic effects [(22)]. Lenalidomide has been demonstrated to possess anti-angiogenic activity through inhibition of bFGF, VEGF and TNF-alpha induced endothelial cell migration [(9)]. The effect of IMiDs on innate cell function in subjects with solid tumours has not yet been investigated.

4.2 Indications and Usage of lenalidomide

Revlimid® is approved in EU in combination with dexamethasone for the treatment of subjects with multiple myeloma that have received at least one prior therapy.

4.3 Adverse Events

Most frequently reported adverse events reported during clinical studies with lenalidomide in oncologic and non-oncologic indications, regardless of presumed relationship to study medication include: anemia, neutropenia, thrombocytopenia and pancytopenia, abdominal pain, nausea, vomiting and diarrhoea, dehydration, rash, itching, infections, sepsis, pneumonia, upper respiratory infection, atrial fibrillation, congestive heart failure, myocardial infarction, chest pain, hypotension, hypercalcemia, hyperglycemia, bone pain, dizziness, syncope, renal failure, dyspnea, pleural effusion, pulmonary embolism, deep vein thrombosis, CVA, convulsions, dizziness, death not specified and fractures.

Complete and updated adverse events are available in the Investigational Drug Brochure and the IND Safety Letters.

4.4 Rationale for combining lenalidomide and gemcitabine

Lenalidomide (Revlimid®) belongs to a proprietary class of Celgene compounds called IMiDs®. IMiDs®, of which thalidomide is the parent compound, have both immunomodulatory and anti-angiogenic properties which could confer antitumour and antimetastatic effects. Lenalidomide has been demonstrated to possess anti-angiogenic activity through inhibition of bFGF, VEGF and TNF-alpha induced endothelial cell migration, due at least in part to inhibition of Akt phosphorylation response to bFGF. [8] In addition, lenalidomide has a variety of immunomodulatory effects. Lenalidomide stimulates T cell proliferation, and the production of IL-2, IL-10 and IFN-gamma, inhibits IL-1 beta and IL-6 and modulates IL-12 production.[10] Upregulation of T cell derived IL-2 production is achieved at least in part through increased AP-1 activity. [3]

Although the exact antitumour mechanism of action of lenalidomide is unknown, a number of mechanisms are postulated to be responsible for lenalidomide's activity

against multiple myeloma. Lenalidomide has been shown to increase T cell proliferation, which leads to an increase in IL-2 and IFN-gamma secretion. The increased level of these circulating cytokines augment natural killer cell number and function, and enhance natural killer cell activity to yield an increase in multiple myeloma cell lysis. [11] In addition, lenalidomide has direct activity against multiple myeloma and induces apoptosis or G1growth arrest in multiple myeloma cell lines and in multiple myeloma cells of subjects resistant to melphalan, doxorubicin and dexamethasone. [11]

Gemcitabine (Gemzar®) is a synthetic pyrimidine nucleoside analogue that is used as standard treatment of advanced pancreatic cancer. Beside the cytotoxic activity of gemcitabine, accumulating evidence has indicated that the product promote specific anticancer immune responses that contribute to the therapeutic effects of conventional therapy. Chemotherapy-induced tumour cell death is likely to increase amounts of antigens loaded on antigen-presenting cells within tumours, providing some of the signals required for T-cell re-stimulation within tumours. Chemotherapy, including gemcitabine, can promote apoptosis by inducing an upregulation of death receptors on tumour cells. Because T-cells can use this pathway to kill targets, this can make the tumour cells more susceptible to T-cell mediated destruction [(13)]. Pre-clinical and clinical data have shown that certain chemotherapy agents, including gemcitabine, can reduce the frequency of circulating CD4+CD25+regulatory T cells (T_{reg}) [(12)]. Moreover, gemcitabine reduces the frequency of myeloid suppressor cells [(14)].

In solid tumours, lenalidomide has been well-tolerated. Moreover, potential clinical efficacy of treatment with lenalidomide has been observed in subjects with advanced disease who have previously received multi-modality treatment when lenalidomide administered as single [(15, 16, 17, 23)] or as combination therapy [(18)]. Down-regulation in survival rate of pancreatic cell lines has more recently been observed, when treated with lenalidomide and gemcitabine in sub-optimal concentrations [(24)]. Moreover, a survival of longer than 33 months of a patient with advanced pancreatic cancer treated with the combination of gemcitabine and lenalidomide, has been reported in a case report [(24)]. Those data supports a hypothesis of a potential hyper-additive affect of the treatments given in combination. Therefore lenalidomide and gemcitabine should be of major interest to explore for combination therapy.

5 Study Objectives and Endpoints

5.1 Phase I Study Objectives

5.1.1 *Primary study objective*

To determine the MTD (Maximum Tolerated Dose) and safety of the regimen lenalidomide and gemcitabine as first-line treatment in subjects with advanced pancreatic cancer.

5.2 Phase II Study Objectives

5.2.1 *Primary study objective*

To evaluate the immunomodulatory effects of lenalidomide in combination with gemcitabine in subjects with advanced pancreatic cancer as first-line treatment.

5.2.2 *Secondary study objective*

To evaluate the safety of lenalidomide in combination with gemcitabine in subjects with advanced pancreatic cancer as first-line treatment.

5.3 Endpoints

5.3.1 *Primary Endpoints*

- Phase I study: To evaluate the MTD and safety (type, frequency, and severity) of adverse events (AEs) and relationship of AEs to lenalidomide and gemcitabine.
- Phase II study: Immunomodulatory effects.

5.3.2 *Secondary Endpoints (phase II)*

- Safety (type, frequency, and severity) of adverse events (AEs) and relationship of AEs to lenalidomide and gemcitabine treatment.
- Progression-free survival.
- Overall survival.
- Survival rate at 12 months.

6 Investigational Plan

6.1 Overall design

This is a phase I/II open-label, multi-center study in patients with advanced pancreatic cancer. The feasibility to administer lenalidomide in combination with gemcitabine as first-line treatment and the MTD of lenalidomide in this combination will be determined in the phase I part. Lenalidomide will be administered once daily for 21 days of a 28 day cycle. 3-4 patients will be enrolled to increased dose-cohorts of lenalidomide (15 mg/day, 20 mg/day and 25 mg/day). Gemcitabine 1000 mg/m2 will be administered as intravenous infusion over 30 minutes, weekly for 3 weeks then rest for 1 week (days 1, 8, 15 of 28).When MTD has been established, the efficacy of the combination will be further evaluated in the phase II part of the study. In the phase II part, every other consecutive included patient will be treated with either lenalidomide (days 1-21 of 28) or gemcitabine (days 1, 8, 15 of 28) as monotherapy, during treatment cycle number 1. From treatment cycle number 2 and further, all subjects in the phase II part of the study will be treated with lenalidomide in combination with gemcitabine.

Subjects will be screened within 28 days prior to cycle 1, day 1 as outlined in section 2 (Schedule of Study Assessments). Patients without clinical evidence of progressive disease at the end of the first cycle of therapy will be allowed to continue treatment.

All subjects will continue on treatment until disease progression, unacceptable toxicity or treatment discontinuation for any other reason and be followed for toxicity (dose adjustments resulting from late-onset toxicity will be evaluated).

Adverse events will be graded using the National Cancer Institute (NCI) Common Toxicity Terminology Criteria for Adverse Events v3.0 (CTCAE v3.0), Appendix 2.

All subjects who discontinue study treatment for any reason will continue to be followed for survival during the Follow-up phase.

6.1.1 Phase I part

For details regarding study related assessment (blood tests, exams etc) see section 2. Lenalidomide (Revlimid®) capsules will be taken orally in the morning each day on days 1-21 of each 28-day cycle. Three subjects will be enrolled into each dose cohort for 15, 20 and 25 mg/day, respectively. Initially 3 patients will be enrolled at the starting dose (Dose-level 1), see Table 1, of lenalidomide (15 mg daily days 1-21 of each 28-day cycle). Gemcitabine (Gemzar®), 1000 mg/m2 in 0.9% sodium chloride will be administered as intravenous infusion over 30 minutes, weekly for 3 weeks then rest for 1 week (days 1, 8, 15 of each 28-day cycle). Enrollment to dose-cohorts Dose-level 2 (20 mg daily days 1-21 of a 28-day cycle) and Dose-level 3 (25 mg of lenalidomide daily days 1-21 of a 28-day cycle) should be hold until the last enrolled patient in the

previous dose-cohort has reach the end of cycle 1, i.e. at least 28 days of cycle 1, respectively.

Nine up to 12 patients will participate in the phase I part of the study. The dose escalation process in the three dose-cohorts of the patients is described in detail below. Additional patients may participate in the phase II part of the study once the MTD has been established in the phase I part of the study. Maximum 30 patients will be included (9-12 in the phase I part and 18-21 in the phase II part).

Following cycle one, therapy with lenalidomide and gemcitabine will continue in 21 days of a 28- day cycle, until disease progression, unacceptable toxicity or the patient chooses to withdraw from therapy.

6.1.1.1 Phase I Dose Escalation Process

- Initially three patients will be enrolled at the starting dose (Dose Level 1), of lenalidomide (15 mg daily days 1-21 of each 28-day cycle), see Table 1. Gemcitabine (Gemzar®), 1000 mg/m2 in 0.9% sodium chloride will be administered as intravenous infusion over 30 minutes, weekly for 3 weeks then rest for 1 week (days 1, 8, 15 of each 28-day cycle).
- If none of the subjects at Dose Level 1 experiences dose-limiting toxicity (DLT), as defined at 6.1.1.3, enrolment for Dose Level 2 will be done (20 mg lenalidomide daily days 1-21 of a 28-day cycle).
- If none of the subjects at Dose Level 2 experiences dose-limiting toxicity (DLT), as defined at 6.1.1.3, enrolment for Dose-level 3 will be done (25 mg of lenalidomide daily days 1-21 of a 28-day cycle).
- Enrollment to dose cohorts Dose Level 2 and Dose Level 3, respectively, should be hold until the last enrolled patient in the previous dose-cohort has reach the end of cycle 1, i.e. at least 28 days of cycle 1.
- If 1 subject experiences DLT, 1 additional subject will be enrolled into the Dose Level.
- If $\geq$ 2 of the 4 subjects encounter DLT, then the MTD has been exceeded and the next lower dose level will be declared to be the MTD.

Table 1. Phase I Dose Cohorts

Dose Level	Lenalidomide Dose Days 1-21 of Each 28-Day Cycle	Gemcitabine Dose Days 1, 8, 15 of Each 28-Day Cycle
1<br>Starting dose	15 mg	1000 mg/m2
2	20 mg	1000 mg/m2
3	25 mg	1000 mg/m2

3-4 subjects will be enrolled into each dose cohort for 15, 20 and 25 mg of lenalidomide daily days 1-21 of a 28-day cycle.

6.1.1.2 Subject Replacement

Phase I part; Subjects, who discontinue study drug prior to the completion of their first 21-day study assessments for a reason other than an adverse event, will be replaced in order to have an adequate number of subjects for determination of the MTD.

Phase II part; Subjects, who received at least two cycles of the defined treatment according to the study protocol is considered to be evaluable. If study treatment discontinued before completion of two treatment cycles for any other reason than AEs as defined as protocol, this subject will be replaced.

6.1.1.3 Phase I Determination of Maximum Tolerated Dose

The maximum tolerated dose (MTD) of lenalidomide given in combination with gemcitabine will be defined as the highest dose level at which no more than 1 out of 4 subjects experiences a DLT.

6.1.1.4 Phase I Definition of Dose Limiting Toxicity

Dose limiting toxicity (DLT) will be assessed continuously, and refers to a medically significant event which meets one of the following criteria using the National Cancer Institute (NCI) Common Toxicity Terminology Criteria for Adverse Events v3.0 (CTCAE v3.0), Appendix 4.

- Inability to deliver all doses on scheduled dose levels during Cycle 1 due to an unexpected drug-related toxicity. (If the gemcitabine-related toxicity is an expected toxicity as per current package insert, then it will not be considered a DLT.) These subjects should be discontinued from the study and started on an alternative, approved therapy at the discretion of the Investigator.
- Inability to deliver the intended doses of lenalidomide in Cycle 1 due to drug-related toxicity as outlined below:

- Any Grade 3 or 4 non-hematological toxicity (excluding alopecia) lasting for ≥ 14 days (the toxicity must NOT resolve/regress to ≤ Grade 1 within 14 days of onset after optimally treated with supportive measures).
- Febrile neutropenia.
- Any Grade 4 neutropenia lasting for ≥ 7 days (the toxicity must NOT resolve/regress to ≤ Grade 1 within 7 days of onset after optimally treated with supportive measures).
- Grade 4 thrombocytopenia.

These subjects should be discontinued from the study and started on an alternative, approved therapy at the discretion of the Investigator.

- The occurrence of one of the above drug-related toxicities will result in a clinical and/or laboratory assessment to be performed within 7 days following the initial finding to examine the subject for resolution of the toxicity. Lack of resolution of any of these toxicities within 14 days according to the guidelines above will be considered a DLT and result in discontinuation of that subject.
- Grade 4 liver enzyme toxicity/abnormality (transaminitis) (serum transaminase > 20 x upper limit of normal (ULN) is a DLT, while Grade 3 transaminitis (serum transaminase > 5 x and ≤ 20 x ULN) must be present for ≥ 7 days to be considered a DLT.
- Exceptions: Grade 3 or 4 venous thromboembolic events are not considered to DLT as long as anticoagulant therapy can be administered.

6.1.2 Phase II part

Additionally 18-21 subjects will be enrolled in the phase II part of the study.

Lenalidomide (Revlimid ®) Lenalidomide at dose determined in Phase I, will be administered orally once daily for 21 days followed by 7 days rest.

Gemcitabine (Gemzar®), 1000 mg/m2 in 0.9% sodium chloride will be administered as intravenous infusion over 30 minutes, weekly for 3 weeks then rest for 1 week (days 1, 8, 15 of 28).

Treatment Arms in Cycle 1:

Every other consecutive included subject in phase II part will be treated with either single lenalidomide (days 1-21 of 28) or single gemcitabine (days 1, 8, 15 of 28) during Cycle 1, see Section 2 and Figure 1. The first included patient in phase II part will start with single lenalidomide.

From treatment cycle number 2 and beyond, all subjects in the phase II part of the study will be treated with lenalidomide in combination with gemcitabine until progression of the disease, unacceptable toxicity or subjects withdrawn.

Figure 1. Schematic presentation of treatment Schedule *

Phase I (n = 9-12):

Days	1	2	3	4	5	6	7	8	9	10	11	12	13	14	15	16	17	18	19	20	21	22	23	24	25	26	27	28	1	2	3	4	5	6	7	8	9	10	11	12	13	14	15	16	17	18	19	20	21	22	23	24	25	26	27	28
Cycle no.	1																												2 and beyond																											
Week	1							2							3							4							5							6							7							8 etc						
Lenalidomide	x	x	x	x	x	x	x	x	x	x	x	x	x	x	x	x	x	x	x	x	x								x	x	x	x	x	x	x	x	x	x	x	x	x	x	x	x	x	x	x	x	x							
Gemcitabine	x							x							x														x							x							x													

Phase II (n=18-21)

Start Lenalidomide group (n =9-11)

Days	1	2	3	4	5	6	7	8	9	10	11	12	13	14	15	16	17	18	19	20	21	22	23	24	25	26	27	28	1	2	3	4	5	6	7	8	9	10	11	12	13	14	15	16	17	18	19	20	21	22	23	24	25	26	27	28
Cycle no.	1																												2 and beyond																											
Week	1							2							3							4							5							6							7							8 etc						
Lenalidomide	x	x	x	x	x	x	x	x	x	x	x	x	x	x	x	x	x	x	x	x	x								x	x	x	x	x	x	x	x	x	x	x	x	x	x	x	x	x	x	x	x	x							
Gemcitabine																													x							x							x													

Start Gemcitabine group (n=9-11)

Days	1	2	3	4	5	6	7	8	9	10	11	12	13	14	15	16	17	18	19	20	21	22	23	24	25	26	27	28	1	2	3	4	5	6	7	8	9	10	11	12	13	14	15	16	17	18	19	20	21	22	23	24	25	26	27	28
Cycle no.	1																												2 and beyond																											
Week	1							2							3							4							5							6							7							8 etc						
Lenalidomide																													x	x	x	x	x	x	x	x	x	x	x	x	x	x	x	x	x	x	x	x	x							
Gemcitabine	x							x							x														x							x							x													

* Low molecular weight heparin (LMWH) at prophylactic dose (e.g. dalteparin 5000 IU subcutaneously daily) to be administered during protocol treatment when platelet count is > 50 x 10^9/L.

6.1.3 Venous thromboembolism (VTE) prophylaxis

All patients should receive low molecular weight heparin (LMWH) at prophylactic dose (e.g. dalteparin 5000 IU subcutaneously daily) during protocol treatment when platelet count is > 50 x 10^9/L. If platelet count is ≤ 50 x 10^9/L, the anticoagulation therapy should be hold until recovery to platelet count is > 50 x 10^9/L.

6.1.4 Procedures for enrolment of eligible patients and patient numbering

Patients who are candidates for enrolment in the study will be evaluated for eligibility by the investigator to ensure that criteria given in section 6.3.1 and 6.3.2 have been satisfied, and that the patient is eligible for this clinical study. A patient eligibility form is provided in the CRF.

Regarding Enrolment to dose cohorts Dose Level 2 and 3 in the phase I part of the study, and enrolment to phase II part, see sections 6.1.1.1 and 6.1.2, respectively.

No patient may begin treatment prior to assignment of a patient number.

The investigator will use the CRF to create a new patient with the assigned number and enter the corresponding number for allocation to the treatment group in the appropriate place on each patient CRF.

Upon signing the informed consent form, the patient is assigned a subject number by the investigator.

Patients in phase I part will be assigned patient number I1 plus initials, I2 plus initials etc.

Patients in phase II part will be assigned patient number IILen1 plus initials, IILen2 plus initials etc (subjects who will receive single lenalidomide in cycle 1) and IIGem1 plus initials, IIGem2 plus initials etc (subjects who will receive single gemcitabine in cycle 1). A patient enrolment and identification list must be maintained by the investigator.

6.2 Investigational Drug

6.2.1 Lenalidomide

6.2.1.1 Lenalidomide Description

REVLIMID® (lenalidomide), a thalidomide analogue, is an immunomodulatory agent with anti-angiogenic properties. The chemical name is 3-(4-amino-1-oxo 1,3-dihydro - 2H-isoindol-2-yl) piperidine-2,6-dione and it has the following chemical structure:

Chemical Structure of Lenalidomide

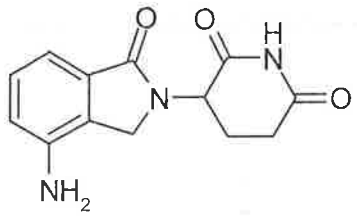

3-(4-amino-1-oxo 1,3-dihydro-2*H*-isoindol-2-yl) piperidine-2,6-dione

The empirical formula for lenalidomide is $C_{13}H_{13}N_3O_3$, and the gram molecular weight is 259.3.

Lenalidomide is off-white to pale-yellow solid powder. It is soluble in organic solvent/water mixtures, and buffered aqueous solvents. Lenalidomide is more soluble in organic solvents and low pH solutions. Solubility was significantly lower in less acidic buffers, ranging from about 0.4 to 0.5 mg/ml. Lenalidomide has an asymmetric carbon atom and can exist as the optically active forms S(-) and R(+), and is produced as a racemic mixture with a net optical rotation of zero.

REVLIMID® (lenalidomide) is available in capsules for oral administration. Each capsule contains lenalidomide as the active ingredient and the following inactive ingredients: lactose anhydrous, microcrystalline cellulose, croscarmellose sodium, magnesium stearate, gelatin, titanium dioxide and black ink. In this study 5, 10, 15, 20 and 25 mg capsules will be used.

6.2.1.2 Clinical Pharmacology

Mechanism of Action:

The mechanism of action of lenalidomide remains to be fully characterized. Lenalidomide possesses immunomodulatory and antiangiogenic properties. Lenalidomide inhibited the secretion of pro-inflammatory cytokines and increased the secretion of anti-inflammatory cytokines from peripheral blood mononuclear cells. Lenalidomide inhibited cell proliferation with varying effectiveness (IC50s) in some but not all cell lines. Of cell lines tested, lenalidomide was effective in inhibiting growth of Namalwa cells (a human B cell lymphoma cell line with a deletion of one chromosome 5) but was much less effective in inhibiting growth of KG-1 cells (human myeloblastic cell line, also with a deletion of one chromosome 5) and other cell lines without chromosome 5 deletions. Lenalidomide inhibited the expression of cyclooxygenase-2 (COX-2) but not COX-1 in vitro.

6.2.1.3 Pharmacokinetics and Drug Metabolism:

Absorption:

Lenalidomide, in healthy volunteers, is rapidly absorbed following oral administration with maximum plasma concentrations occurring between 0.625 and 1.5 hours post-dose. Co-administration with food does not alter the extent of absorption (AUC) but does reduce the maximal plasma concentration (Cmax) by 36%. The pharmacokinetic disposition of lenalidomide is linear. Cmax and AUC increase proportionately with increases in dose. Multiple dosing at the recommended dose-regimen does not result in drug accumulation.

Pharmacokinetic sampling in myelodysplastic syndrome (MDS) subjects was not performed. In multiple myeloma subjects maximum plasma concentrations occurred between 0.5 and 4.0 hours post-dose both on Days 1 and 28. AUC and Cmax values increase proportionally with dose following single and multiple doses. Exposure (AUC) in multiple myeloma subjects is 57% higher than in healthy male volunteers.

Pharmacokinetic Parameters:

Distribution:

In vitro (14C)-lenalidomide binding to plasma proteins is approximately 30%.

Metabolism and Excretion:

The metabolic profile of lenalidomide in humans has not been studied. In healthy volunteers, approximately two-thirds of lenalidomide is eliminated unchanged through urinary excretion. The process exceeds the glomerular filtration rate and therefore is partially or entirely active. Half-life of elimination is approximately 3 hours.

6.2.1.4 Supplier(s)

Celgene Corporation will supply Revlimid®, lenalidomide free of charge during the study.

6.2.1.5 Dosage form

Lenalidomide will be supplied as 5, 10, 15, 20 and 25 mg capsules for oral administration.

6.2.1.6 Packaging

Drug will be shipped to the pharmacy at the study site in individual bottles. Bottles will contain a sufficient number of capsules to last for 21 days of dosing during a 28 days cycle. Study drug must be dispensed in the original packaging with the label clearly visible. Only one 28 day supply (for 21 days dosing) may be provided to the subject each cycle.

6.2.1.7 Labeling

Lenalidomide investigational supplies are dispensed to the subject's in individual bottles of capsules. Each bottle will identify the contents as study medication.

6.2.1.8 Receipt of study drug

The Investigator or designee is responsible for taking an inventory of each shipment of study drug received, and comparing it with the accompanying study drug accountability form. The Investigator will verify the accuracy of the information on the form, sign and date it and retain a copy in the study file.

6.2.1.9 Storage

At the study site, all investigational study drugs will be stored in a locked, safe area to prevent unauthorized access.

The study drug should be stored at room temperature away from direct sunlight and protected from excessive heat and cold.

6.2.1.10 Unused study drug supplies

Investigator or designee will return unused study drugs to pharmacy for destruction. If any study drug is lost or damaged, its disposition should be documented in the source documents. Subjects will be instructed to return empty bottles or unused capsules.

6.2.2 Gemcitabine

6.2.2.1 Gemcitabine description

Gemcitabine (Gemzar®) is a synthetic pyrimidine nucleoside analogue that is used as standard treatment of advanced pancreatic cancer. Beside the cytotoxic activity of gemcitabine, accumulating evidence has indicated that the product promote specific anticancer immune responses that contribute to the therapeutic effects of conventional therapy [12-14].

6.2.2.2 Dosing and administration

Gemcitabine, 1000 mg/m2 in 0.9% sodium chloride will be administered as intravenous infusion over 30 minutes, weekly for 3 weeks then rest for 1 week.

6.2.2.3 Supplier, packaging and labeling

Gemzar® will be supplied according to standard health care procedures from the pharmacy, as the trial is conducted within its approved pancreatic cancer indication.

6.3 Screening and Eligibility

The Investigator is responsible for keeping a record of all subjects who sign an Informed Consent Form for entry into the study. All subjects will be screened for eligibility. Screening procedures are outlined in Section 2, Schedule of Study Assessments and unless otherwise specified, must take place within 28 days prior to initiation of therapy.

Subjects must meet the following inclusion/exclusion criteria to be eligible for the study.

6.3.1 Inclusion Criteria

Subjects must meet all of the following inclusion criteria to be eligible for enrolment into the study:

1. Histologically or cytologically confirmed, unresectable, locally advanced, or metastatic adenocarcinoma of the pancreas.
2. ECOG performance status of 0 or 1, see Appendix 1.
3. Life expectancy > 12 weeks.
4. Must understand and voluntarily sign an informed consent form.
5. Age > 18 years at the time of signing informed consent form.
6. Must be able to adhere to the study visit schedule and other protocol requirements.
7. Female subjects of childbearing potential† must:
    a. Understand that the study medication is expected to have a teratogenic risk
    b. Agree to use, and be able to comply with, effective contraception without interruption, 4 weeks before starting study drug, throughout study drug therapy (including dose interruptions) and for 4 weeks after the end of study drug therapy, even if she has amenorrhoea. This applies unless the subject commits to absolute and continued abstinence confirmed on a monthly basis. The following are effective methods of contraception*
        i. Implant**
        ii. Levonorgestrel-releasing intrauterine system (IUS)**
        iii. Medroxyprogesterone acetate depot
        iv. Tubal sterilization

† A female subject or a female partner of a male subject is considered to have childbearing potential unless she meets at least one of the following criteria: Age ≥50 years and naturally amenorrhoeic for ≥ 1 year (amenorrhea following cancer therapy does not rule out childbearing potential), premature ovarian failure confirmed by a specialist gynecologist, previous bilateral salpingo-oophorectomy or hysterectomy, XY genotype, Turner syndrome or uterine agenesis.

v. Sexual intercourse with a vasectomised male partner only; vasectomy must be confirmed by two negative semen analyses

vi. Ovulation inhibitory progesterone-only pills (i.e., desogestrel)

If not established on effective contraception, the female subject must be referred to an appropriately trained health care professional for contraceptive advice in order that contraception can be initiated.

* Combined oral contraceptive pills are not recommended. If a subject was using combined oral contraception, she must switch to one of the methods above. The increased risk of VTE continues for 4 to 6 weeks after stopping combined oral contraception.

** Prophylactic antibiotics should be considered at the time of insertion particularly in subjects with neutropenia due to risk of infection. Copper-releasing intrauterine devices are generally not recommended due to the potential risks of infection at the time of insertion and menstrual blood loss which may compromise patients with neutropenia or thrombocytopenia.

c. Agree to have a medically supervised pregnancy test with a minimum sensitivity of 25 mIU/ml on the day of the study visit or in the 3 days prior to the study visit once the subject has been on effective contraception for at least 4 weeks. This requirement also applies to women of childbearing potential who practice complete and continued abstinence. The test should ensure the subject is not pregnant when she starts treatment.

d. Agree to have a medically supervised pregnancy test every 4 weeks including 4 weeks after the end of study treatment, except in the case of confirmed tubal sterilization. These pregnancy tests should be performed on the day of the study visit or in the 3 days prior to the study visit. This requirement also applies to women of childbearing potential who practice complete and continued abstinence.

e. Understand that even if she has amenorrhea, she must follow all the advice on effective contraception.

f. She understands the potential consequences of pregnancy and the need to rapidly consult if there is a risk of pregnancy.

8. Male subjects must:

   a. Agree to use condoms throughout study drug therapy, during any dose interruption and for one week after cessation of study therapy if their partner is of childbearing potential and has no contraception.

   b. Agree not to donate semen during study drug therapy and for one week after end of study drug therapy.

9. All subjects must:

   a. Agree to abstain from donating blood while taking study drug therapy and for one week following discontinuation of study drug therapy.
   b. Agree not to share study medication with another person and to return all unused study drug to the investigator

6.3.2 Exclusion criteria

The presence of any of the following will exclude a subject from study enrolment:

1. Prior use of systemic chemotherapy for the treatment of adenocarcinoma of the pancreas (with the exception of gemcitabine, fluorouracil, or capecitabine in the adjuvant setting).
2. Any of the following laboratory abnormalities:
   a. Absolute neutrophil count (ANC) <1,5 x 10^9/L
   b. Platelet count <100 x 10^9/L
   c. Serum creatinine >2,0 mg/dL (SI units > 177 µmol/L)
   d. Serum-AST or ALT >3 x upper limit of normal (ULN); in case of liver metastases > 5 x ULN.
   e. Serum total bilirubin > 3 x ULN
   f. Haemoglobin < 90 g/L
3. Prior history of malignancy within 5 years (except basal or squamous cell carcinoma or carcinoma in situ of the cervix or breast, localized prostate cancer with PSA < 1,0 mg/dL).
4. Subjects with a history of or active DVT or PE that are not therapeutically managed on a stable dose of appropriate anticoagulant.
5. Brain metastases (subjects that are asymptomatic and do not require steroid control may be enrolled at the discretion of the investigator).
6. Surgery within 28 days prior to cycle 1 Day 1 (minimally invasive procedures for the purpose of diagnosis or staging of the disease are permitted, including stent placement and insertion of central venous access advice).
7. Any condition, including the presence of laboratory abnormalities, which places the subject at unacceptable risk if he/she were to participate in the study or confounds the ability to interpret data from the study.
8. Any serious medical condition or psychiatric illness that places the subject at an unacceptable risk for study participation or would prevent the subject from signing the informed consent form.
9. Prior therapy with lenalidomide or thalidomide.

10. Use of any other experimental drug or therapy within 28 days prior to Cycle 1 Day 1.
11. Pregnant or lactating females.

6.4 Visit schedule and assessments

Screening Assessments and all on study scheduled visits and assessments are outlined in Section 2, Schedule of Study Assessments.

For females of child bearing potential, counseling about pregnancy precautions and the potential risks of fetal exposure must be conducted at a minimum of every 28 days. During counseling, subjects must be reminded to not share study drug and to not donate blood.

Pregnancy testing and counseling must be performed if a subject misses her period or if her pregnancy test or her menstrual bleeding is abnormal. Study drug treatment must be discontinued during this evaluation.

In addition to the required pregnancy testing, the Investigator must confirm with FCBP that she is continuing to use a reliable method of birth control at each visit.

Counseling for all men about the requirement for latex condom use during sexual contact with females of childbearing potential and the potential risks of fetal exposure must be conducted at a minimum of every 28 days. During counseling, subjects must be reminded to not share study drug and to not donate blood, sperm, or semen.

All subjects taking lenalidomide must be counseled about pregnancy prevention and the risk of fetal exposure every 28 days.

An unscheduled visit can occur at any time during the study. Source must be maintained for these unscheduled visits. The date for the visit and any data generated must be recorded on the appropriate CRF. Source documents for these unscheduled visits must also be maintained.

At treatment discontinuation, subjects will undergo off study evaluations per the Schedule of Study Assessments, Section 2. In addition, a safety assessment will be done approximately 28 days post the last dose of study drug.

6.5 Drug Administration

6.5.1 Treatment assignments

See section 6.1.1 and 6.1.2.

6.5.2 Dosing regimen

See section 6.1.

6.5.3 Record of administration

Accurate records will be kept of all study drug administration (including dispensing and dosing) will be made in the source documents.

6.6 Dose Continuation, Modification and Interruption

Subjects will be evaluated for AEs at each visit with the NCI CTCAE v3.0 (see Appendix 2) used as a guide for the grading of severity. Refer to Sections 6.6.3.and 6.6.4 for instructions on initiation of a new cycle of therapy and dose modifications during a cycle of therapy.

6.6.1 Instructions for dose continuation, modification and interruption

Phase I: In cycle 1, no dose modification is permitted for lenalidomide in any dose cohort. If the subject experience drug-related toxicity during cycle 1, the gemcitabine dose will be modified 1 dose level (-1) at day 8, see Table 2. If AE not restored to grade <=2 within 7 days dose reduction to (-2) levels of Gemcitabine is permitted at day 15 if (see table 2). Critical DLTs (those triggering an End-of-Cycle-1 DLT rate assessment) will be those occurring within the first cycle of treatment not resolving to a <= Grade 3.

Table 2.

Dose Level	Lenalidomide Dose Days 1-21 of Each 28-Day Cycle	Gemcitabine Dose Days 1, 8, 15 of Each 28-Day Cycle
-2	5 mg	500 mg/m2
-1	10 mg	750 mg/m2
**1 Starting Dose**	15 mg	1000 mg/m2
2	20 mg	1000 mg/m2
3	25 mg	1000 mg/m2

In subsequent cycles, (cycle 2 and further), dose modifications of lenalidomide and gemcitabine will be made for toxicity occurring during a cycle, or newly encountered on Day 1 of a new cycle as outlined in Table 3 and 4.

Phase II; In cohort of patients receiving single lenalidomide in cycle 1, dose reductions of lenalidomide are permitted according to defined schedule in tables 3 and 4. In subsequent cycles dose reductions of both lenalidomide and gemcitabine are permitted as above.

In cohort of patients receiving single gemcitabine in cycle 1, dose reductions of gemcitabine are permitted according to defined schedule in tables 3 and 4. In subsequent cycles dose reductions of both lenalidomide and gemcitabine are permitted as above.

6.6.2 Dose reduction steps for Lenalidomide and gemcitabine

Dose modifications of Lenalidomide and gemcitabine will be made for toxicity occurring during a cycle, or newly encountered on Day 1 of a new cycle as outlined in Table 3 and 4.

6.6.3 Instructions for dose modifications or interruption during a cycle

During a cycle, a WBC with differential counting and platelet count will be checked on Day 7 or 8 and Day 14 or 15 prior to the administration of the gemcitabine dose and dose modification will be implemented as outlined in Table 3 and Table 4. Dose modification recommendations listed below are general guidelines, and appropriate dose adjustments for patient safety, tolerability and therapeutic effect should be done if needed after approval by the Principal Investigator or her representative.

Table 3. Dosing adjustment Guidelines for Drug-related hematologic toxicities During a Cycle

Hematologic toxicity	DAY of cycle	Lenalidomide	Gemcitabine
Neutrophils ≥ 1.0 x 10^9/L and Platelets > 75 x 10^9/L	Day 8	No change from Day 1	No change from Day 1
	Day 15	No change from Day 1 unless: if Day 8 held, reduce by one dose level	No change from Day 1 unless: If Day 8 held, reduce by one dose level
Neutrophils = 0.9-0.75 x 10^9/L or Platelets = 75– 50 x 10^9/L	Day 8	Reduce by one dose level	Reduce by one dose level
Neutrophils = 0.9-0.75 x 10^9/L or Platelets = 75– 50 x 10^9/L	Day 15	Reduce by one level unless: if Day 8 already reduced, maintain reduced dose.	Reduce by one level unless: if Day 8 already reduced, maintain reduced dose.
Neutrophils <0.75 x 10^9/L or Platelets < 50 x 10^9/L	Day 8	HOLD and re-asses on day 15	HOLD and re-asses on day 15
	Day 15	HOLD	HOLD
Febrile neutropenia (Defined as ANC<1.0x10^9/L and fever ≥ 38.5°C)	Any day	HOLD; reduce by one dose level upon recovery to Neutrophils ≥ 1.0 x 10^9/L.	HOLD; reduce by one dose level at next scheduled treatment upon recovery to Neutrophils ≥ 1.0 x 10^9/L

Table 4. Dosing adjustment Guidelines for Non-Hematologic Toxicity During a Cycle

Non-Hematologic toxicity	DAY of cycle	Lenalidomide	Gemzar
Non blistering rash Grade 1 or 2	Any day	No change	No change
Grade 3		Hold until resolved to < Grade 2 and reduce by one dose level.	Hold until resolved to < Grade 2. Resume next scheduled dose at one dose level reduction.
Grade 4		Discontinue therapy	HOLD until resolved and may continue therapy off protocol at investigators discretion.
Desquamating (blistering) rash or Erythema multiforme any grade.	Any day	Discontinue therapy	HOLD until resolved and may continue therapy off protocol at investigators discretion.
Allergic reaction or hyper sensitivity assessed as lenalidomide related Grade 2/3.		HOLD until resolved to ≤ Grade 1, resume at one dose level.	HOLD until resolved to Grade ≤ 1. Maintain dose.
Grade 4		Discontinue therapy	HOLD until resolved and may continue therapy off protocol at investigators discretion.
Sinus bradycardia/ other cardiac arrhythmia Grade 2	Any day	HOLD until resolves to ≤ Grade 1, reduce one dose level.	HOLD until resolved to ≤ Grade 1. Maintain dose at investigators discretion.
≥ Grade 3		Discontinue therapy	HOLD until resolved and may continue therapy off protocol at investigators discretion.
Venous thrombosis/embolism	Any day	Hold (interrupt) dose and start full anticoagulation; restart at investigator's discretion (maintain dose level). If subject is an therapeutic dose of anticoagulation, discontinue lenalidomide.	Hold (interrupt) dose and start full anticoagulation; restart at investigator's discretion (maintain dose level).
Asymptomatic Hyperthyroidism or hypothyroidism	Any day	Evaluate etiology, and initiate appropriate therapy.	Maintain dose at investigators discretion.
Symptomatic Hyperthyroidism or hypothyroidism		Hold until symptoms resolved and appropriate treatment implemented.	
Other non-hematologic toxicity assessed as lenalidomide related ≥ Grade 3	Any day	HOLD until resolved to ≤ Grade 2. Restart with one dose level reduction.	Maintain dose or hold at investigators discretion.
Other non-hematologic (renal/hepatic) toxicity assesses as Gemcitabine related ≥ Grade 3	Any day	Hold at investigators discretion.	HOLD until resolved to ≤ grade 2. Restart with one dose level reduction.

- If therapy on day 8 is reduced or held, day 15 may commence at the reduced dose if recovery is adequate for treatment (only one reduction required per cycle if recovery is adequate).
- If multiple toxicities occur, the dose reductions to be implemented during a cycle, should be the greatest required for any given toxicity (multiple reductions are not required for multiple toxicities on a given day).

6.6.4 Dosing adjustment Guidelines for initiation of a new cycle of therapy

The dose of lenalidomide and gemcitabine to be implemented is based on the toxicity and dose reduction encountered in the previous cycle as listed in Table 5:

Table 5. Dose determination of Lenalidomide and gemcitabine for initiation of a new cycle

Day of Cycle	Day 8	Day 15	Next cycle if recovered by Day 1	Next cycle if recovery delayed to within 14 days of scheduled Day 1	Next cycle not recovered within 14 days of scheduled Day 1
If dose was	Held	Held	Reduce	Reduce	discontinue
If dose was	Reduced	Held	Same as Day 8	Same as Day 8	discontinue
If dose was	Held	Reduced	Same as Day 15	Same as Day 15	discontinue
If dose was	Reduced	Same as Day 8	Same as Day 8* * may also consider full dose	Same as Day 8	discontinue
If dose was	Full dose	Reduced	Full dose	reduce	discontinue
If dose was	Full dose	Full dose	Full dose	reduce	discontinue

A new cycle of therapy may begin on a scheduled Day 1 if the following conditions are met on day -1 to day 1:

- The Neutrophils $\geq$ 1.0 x 109/L
- The platelet count is $\geq$ 75 x 109/L
- Any lenalidomide-related allergic reaction/hypersensitivity or sinus bradycardia/ other cardiac arrhythmia adverse event that may have occurred has resolved to $\leq$ grade 1 severity;

- Any other lenalidomide/gemcitabine related adverse event that may have occurred has resolved to ≤ grade 2 severity.

If these conditions are not met on Day 1 of a new cycle, the subject will be evaluated weekly and a new cycle of therapy will not be initiated until the toxicity has resolved as described above. If therapy can not be initiated within 14 days of a scheduled Day 1, therapy will be discontinued.

6.6.5 Treatment compliance

At all times, when dispensing study drug, research center personnel will review the instructions, printed on the packaging, with subjects. Subjects will be asked to maintain a diary to record the drug administration. Subjects will be asked to bring any unused study drug to the research center at their next visit. Research personnel will count and record the number of used and unused study drug capsules at each visit and reconcile with the subject diary.

6.7 Concomitant therapy

6.7.1 Recommended concomitant therapy

- Subjects should receive full supportive care, including transfusions of blood and blood products, antibiotics, and antiemetics when appropriate.
- The use of growth factors is permitted after Cycle 1 in Phase I and anytime during Phase II, however should not be routinely used as prophylaxis to avoid dose reductions or delays.

6.7.2 Prohibited concomitant therapy

Concomitant use of chemotherapy, radiation, thalidomide, or other investigational agents is not permitted while subjects are receiving study drug during the treatment phase of the study.

6.8 Discontinuation of Study Treatment

Treatment will continue until the occurrence of any of the following events.

- Progressive disease.
- Adverse event(s) that, in the judgment of the Investigator, may cause severe or permanent harm or which rule out continuation of study drug.
- Major violation of the study protocol.
- Withdrawal of consent.
- Lost to follow up.

- Death.
- Suspected pregnancy.

6.9 Follow-Up

See Section 2 for details for follow-up. Subjects, who discontinue treatment for any reason, will be followed up according to doctor's decision. At treatment discontinuation, subjects will undergo a safety assessment approximately 28 days post the last dose of study drug. In addition off study evaluations per the Schedule of Study Assessments, Section 2 will be done.

Subjects, who are discontinued from protocol treatment due to unacceptable toxicity, will be followed for 30 days or until resolution of treatment related toxicity.

7 Adverse events

7.1 Adverse Event

An adverse event (AE) is any noxious, unintended, or untoward medical occurrence occurring at any dose that may appear or worsen in a subject during the course of a study. It may be a new intercurrent illness, a worsening concomitant illness, an injury, or any concomitant impairment of the subject's health, including laboratory test values (as specified by the criteria below), regardless of aetiology. Any medical condition that was present prior to study treatment and that remains unchanged or improved should not be recorded as an AE. If there is a worsening of that medical condition this should be considered an AE. A diagnosis or syndrome should be recorded on the AE page of the Case Report Form rather than the individual signs or symptoms of the diagnosis or syndrome.
All AEs will be recorded by the Investigator(s) during the period between start of the first cycle (Day 1) until 30 days following the last dose of study drug administration (gemcitabine/ lenalidomide).

7.1.1 Abnormal laboratory values defined as adverse events

An abnormal laboratory value is considered to be an AE if the laboratory abnormality is characterized by any of the following:

- Results in discontinuation from the study.
- Requires treatment, modification/interruption of study drug dose, or any other therapeutic intervention.
- Is judged by the Investigator(s) to be of significant clinical importance.

If a laboratory abnormality is one component of a diagnosis or syndrome, then only the diagnosis or syndrome should be recorded on the AE page of the CRF. If the abnormality was not a part of a diagnosis or syndrome, then the laboratory abnormality should be recorded as the AE.

7.2 Serious adverse event

A serious adverse event (SAE) is any AE which:

- Results in death
- Is life-threatening (i.e., in the opinion of the Investigator(s) the subject is at immediate risk of death from the AE)
- Requires in subject hospitalization or prolongation of existing hospitalization

- Results in persistent or significant disability/incapacity (a substantial disruption of the subject's ability to conduct normal life functions)
- Is a congenital anomaly/birth defect
- Constitutes an important medical event

Important medical events are defined as those occurrences that may not be immediately life threatening or result in death, hospitalization, or disability, but may jeopardize the subject or require medical or surgical intervention to prevent one of the other outcomes listed above. Medical and scientific judgment should be exercised in deciding whether such an AE should be considered serious.

Events not considered to be SAEs are hospitalizations which: were planned before entry into the clinical study; are for elective treatment of a condition unrelated to the studied indication or its treatment; occur on an emergency outsubject basis and do not result in admission (unless fulfilling other criteria above); are part of the normal treatment or monitoring of the studied indication and are not associated with any deterioration in condition.

If an AE is considered serious, both the AE pages of the CRF and the SAE Report Form (Appendix 4) must be completed.

For each SAE, the Investigator(s) will provide information on severity, start and stop dates, relationship to study drug, action taken regarding study drug, and outcome.

7.2.1 Pregnancies

7.2.1.1 Female of Childbearing Potential:

Pregnancies and suspected pregnancies (including a positive pregnancy test regardless of age or disease state) of a female subject occurring while the subject is on study drug, or within 28 days of the subject's last dose of study drug, are considered events to be reported immediately to Sponsor and Celgene. If the subject is on study drug, the study drug is to be discontinued immediately and the subject instructed to return any unused portion of the study drug to the Investigator. The pregnancy, suspected pregnancy, or positive pregnancy test must be reported to Sponsor who will inform Celgene immediately by phone then by facsimile using an SAE Report Form, Appendix 4.

The female should be referred to an obstetrician/gynecologist experienced in reproductive toxicity for further evaluation and counseling.

The Investigator will follow the female subject until completion of the pregnancy, and must notify the Sponsor and Celgene of the outcome of the pregnancy within 5 days or as specified below. The Investigator will provide this information as a follow-up to the initial pregnancy report.

If the outcome of the pregnancy meets the criteria for immediate classification as a SAE (i.e., spontaneous or therapeutic abortion [any congenital anomaly detected in an

aborted fetus is to be documented], stillbirth, neonatal death, or congenital anomaly [including that in an aborted fetus]), the Investigator should follow the procedures for reporting SAEs. In the case of a live "normal" birth, the Sponsor and Celgene should be advised as soon as the information is available.

All neonatal deaths that occur within 28 days of birth should be reported, without regard to causality, as SAEs. In addition, any infant death after 28 days that the Investigator suspects is related to the in utero exposure to the study drug should also be reported.

7.2.1.2 Male Subject:

Female partners of males taking investigational product should be advised to call their healthcare provider immediately if they get pregnant. The male subject should notify the Investigator of his partner's pregnancy and her healthcare provider information. The Investigator will then provide this information to the Sponsor and Celgene for follow-up as necessary.

7.3 Classification of severity

For both AEs and SAEs, the investigator(s) must assess the severity of the event. The severity of adverse events (AEs) will be graded on a scale of 1 to 5 according to the National Cancer Institute (NCI) Common Terminology Criteria for Adverse Events Version 3.0 (NCI CTCAE). The NCI CTCAE V3.0 can be viewed on-line at the following NCI web site: http://ctep.cancer.gov/reporting/ctc.html. If a specific event is not included in the NCI CTCAE toxicity scale, the following scale should be used to grade the event.

Grade	Definition
1	**Mild** Awareness of sign, symptom, or event, usually transient, requiring no special treatment and generally not interfering with usual daily activities
2	**Moderate** Discomfort that causes interference with usual activities; usually ameliorated by basic therapeutic manoeuvres
3	**Severe** Incapacitating with inability to do usual activities or significantly affects clinical status and warrants intervention. Hospitalization may or may not be required
4	**Life-threatening** Immediate risk of death; requires hospitalization and clinical intervention.
5	**Death**

7.4 Classification of Relationship/Causality of adverse events (SAE/AE) to study drug

The Investigator(s) must determine the relationship between the administration of study drug and the occurrence of an AE/SAE as Not Suspected or Suspected as defined below:

Not suspected:	The temporal relationship of the adverse event to study drug administration makes a causal relationship unlikely or remote, or other medications, therapeutic interventions, or underlying conditions provide a sufficient explanation for the observed event
Suspected:	The temporal relationship of the adverse event to study drug administration makes a causal relationship possible, and other medications, therapeutic interventions, or underlying conditions do not provide a sufficient explanation for the observed event.

7.5 Serious Adverse Event (SAE) Reporting

7.5.1 Immediate reporting by Investigator to Sponsor and Sponsor reporting to Celgene

The investigator will inform the sponsor of all SAEs within 24 hours in order that the sponsor can fulfil his regulatory reporting obligations within the required timeframes.

This applies to all SAEs, regardless of relationship to the study medication, that occur during the study, those made known to the Investigator(s) within 30 days after a subject's last dose of study drug, and those made known to the investigator(s) at any time that are suspected of being related to the study medication. This must be documented on an SAE form. This form must be completed in English and supplied to Celgene Nordic Drug Safety by the sponsor within 24 hours/1 business day or at the latest on the following working day of being made aware of the event regardless of whether or not the event is listed in the reference document (e.g IB, SmPC). The initial report must be as complete as possible, including details of the current illness and serious adverse event, and an assessment of the causal relationship between the event and the investigational product(s). Information not available at the time of the initial report (e.g., an end date for the adverse event or laboratory values received after the report) must be documented on a follow-up SAE form, Appendix 4.

All adverse event reports must include the patient number, age, sex, severity of reaction, relationship to study drug, date and time of administration of test medications and all concomitant medications, and medical treatment provided.

The investigator must keep copies of all AE information, including correspondence with Celgene, Regulatory Authority and Ethics Committee.

The sponsor will provide Celgene with a copy of the annual safety report at the time of the submission to the regulatory authority and the Ethics Committee.

Contact details for Sponsor:
Prof. Håkan Mellstedt MD, PhD.
Karolinska University Hospital / Institute
Department of Oncology-Pathology
SE-171 76 Stockholm
Sweden
Phone: + 46 8 51774641
Phone: + 46 8 51774308 (secretary)
Fax: + 46 8 318327
e-mail: hakan.mellstedt@karolinska.se

Contact details for Drug Safety Nordic:
Celgene AB
Kista Science Tower
164 51 Kista
Sweden
Phone: + 46 8 7031631
Fax: + 46 8 7061603
E-mail: drugsafety-nordic@celgene.com

7.5.2 Reporting to Regulatory Authorities and the Ethics Committee

The sponsor will inform relevant Regulatory Authorities and the Ethics Committee:

- of all relevant information about serious unexpected adverse events suspected to be related to the study medication that are fatal or life threatening as soon as possible, and in any case no later than seven days after knowledge of such a case. Relevant follow-up information for these cases will subsequently be submitted within an additional eight days.
- of all other serious unexpected events suspected to be related to the study medication as soon as possible , but within a maximum of fifteen days of first knowledge by the investigator.

The sponsor will inform relevant Regulatory Authorities and the Ethics Committee of adverse drug reactions that are Serious, Unlisted/unexpected, and at least possibly associated to the drug (SUSARs), and that have not previously been reported in the Investigators brochure, or reference safety information document. The Regulatory Authority should be informed in a COMS-paper to the Pharmacovigilance Unit,

Medical Producrs Agency, PO BOX 26, S-751 03, Uppsala, Sweden. A clear description of the suspected reaction should be provided along with an assessment as to whether the event is drug or disease related.

Even for gemcitabine the sponsor are obliged to report as above, and the documents used to determine expectedness is the Summary of Product Characteristics (SmPC) [14]

7.6 Adverse event updates

Celgene shall notify the principle investigator/sponsor of the following information:

- Any AE associated with the use of study drug or in other studies that is both serious and unexpected.
- Any finding from tests in laboratory animals that suggests a significant risk for human subjects including reports of mutagenicity, teratogenicity, or carcinogenicity.

The principle investigator/sponsor will forward this information to other investigators involved in the trial.

The sponsor shall notify the EC and the relevant regulatory authorities of any new significant risks to subjects as required.

8 Assessment of study objectives

8.1 Assessment of safety

See section 7.

8.2 Assessment of Immunomodulation

The primary objective of this trial is to ascertain whether treatment with lenalidomide or lenalidomide in combination with gemcitabine induces modulation of immune effector functions and to characterize the nature of immune functions. Phenotyping of T cells (CD4, CD8), NK cells (CD16/CD56) and functional T cell assays will be applied to demonstrate immunomodulating effects. The efficacy of the applied treatment in reducing regulatory T cells (T_{reg}) and on myeloid suppressor cells (MSC) will be monitored in the blood to demonstrate the necessity of reduction of self tolerance for optimal treatment effect. The final goal of the study is to demonstrate immunomodulating effect of lenalidomide as single treatment in patients with pancreatic cancer as compared to gemcitabine alone and to investigate the effect on immune response when lenalidomide and gemcitabine are used in combination.

The following types of Immunomonitoring will be performed (Appendix 3);

- Measurement of T_{reg} frequencies in peripheral blood (CD4/CD25, FoxP3) by FACS and measurement of MSC in blood (CD3, CD11b, CD14, CD15, CD19, CD33, HLA-DR).
- Peripheral blood mononuclear cell (PBMC) analysis:
  - Examination of peripheral blood lymphocyte subpopulations by phenotyping of CD4 and CD8 memory/activated T-cells using CCR7, CD45RA, CD25, CD 69 (by FACS).
  - Assessment of NK/NKT cells in peripheral blood by phenotyping CD56, CD16, CD3 (by FACS).
  - Examination of activated B-cells in peripheral blood by phenotyping CD86, CD80, CD83, HLA-DR by FACS.
  - Assessment of frequencies of immune effector cells in vivo by phenotyping granzyme B and perforin content in cytotoxic T-cells and NK-cells (by FACS).
  - Measurement of intracellular IFNγ in CD4/CD8 T-cells and CD56/CD16 NK-cells using FACS.

- T-cell function:
    - T-cell function will be assessed by Lymphoproliferative Assay (semi-quantitative) using a standard [3H] thymidine-incorporation assay (Appendix 4) in the presence of routinely used antigens and superantigens.

- Cytokine/chemokine production of lymphocytes (Multiplex Assay) (quantitative).
    - Secretion of soluble cytokines/chemokines (IL-6, IL-2, IL-10, GM-CSF, IL-4, IL-5, IL-1 beta, TNFalfa), assessed by Multiplex Assay will give information about the quality of the response (pro-inflammatory, Th_1 vs. Th_2 profile).

Samples for immunomonitoring measurements will be taken at defined intervals, as illustrated in the Study Flow Chart (See section 2).

The in vitro immune responses are monitored 3 time-points in total, see section 2; before treatment (baseline), at the end of treatment with lenalidomide or gemcitabine alone and after treatment with the combination of lenalidomide and gemcitabine.

Collection and processing of samples:

Fresh PBMC for immunomonitoring will be used for all assays described above. Supernatants from the proliferation assays will be frozen and storaged at CCK until measurements of soluble cytokine/chemokine by Multiplex assay. For each subject, 5 mL of plasma will be frozen and storaged.

8.3 Assessment of clinical efficacy

8.3.1 Following parameters will be described, according to section 10:

- Progression free survival
- Overall survival
- Survival rate at 12 months
- Radiological examination by CT scanning of the abdomen and thorax (or Chest X-ray) will be performed at baseline and thereafter every second month as routine.

8.4 Protocol amendments

Any amendment to this protocol must be agreed to by the Principal Investigator and reviewed by Celgene. Written verification of EC approval will be obtained before any amendment is implemented.

8.5 Protocol deviations

When an emergency occurs that requires a deviation from the protocol for a subject, a deviation will be made only for that subject. A decision will be made as soon as possible to determine whether or not the subject (for whom the deviation from protocol was effected) is to continue in the study. The subject's medical records will completely describe the deviation from the protocol and state the reasons for such deviation. In addition, the Investigator will notify the EC in writing of such deviation from protocol.

Non-emergency minor deviations from the protocol will be permitted with approval of the Principal Investigator. No waivers of inclusion and exclusion criteria from the protocol will be permitted.

9 Data Management

9.1 Analyses and Reporting

Safety data will be analyzed continuously and reported and immunomodulatory efficacy data will be analyzed after completion of the phase I and II parts of the study, respectively. All subsequent data collected will be analyzed and reported in a follow-up clinical report.

9.2 Study monitoring and auditing

9.2.1 Investigator responsibilities

Investigator responsibilities are set out in the ICH guideline for Good Clinical Practice (GCP).

Investigators must enter study data onto CRFs or other data collection system. The Investigator will permit study-related monitoring visits and audits, EC review, and regulatory inspection(s), providing direct access to the facilities where the study took place, to source documents, to CRFs, and to all other study documents.

The Investigator, or a designated member of the Investigator's staff, must be available at some time during monitoring visits to review data and resolve any queries and to allow direct access to the subject's records (e.g., medical records, office charts, hospital charts, and study related charts) for source data verification. The data collection must be completed prior to each visit and be made available to the monitor.

10 Biostatistical Analysis

10.1 Overview

The primary objective of the Phase I part is to determine the MTD of lenalidomide when given in combination with gemcitabine to patients with advanced pancreatic cancer, as first line therapy. In the phase II part to describe immunomodulatory effects data by assessment of biomarkers. The primary analysis of immunological response will be performed after the subjects in phase II part have been treated for two cycles. All data will be presented in a descriptive manner within each treatment group.

10.2 Datasets to be analyzed

10.2.1 Primary endpoint (phase I)

Frequency, intensity and duration of AE and SAE will be subject to descriptive analysis.

10.2.2 Primary endpoint (phase II)

Assessment of immunological reaction will be presented in a descriptive manner within each treatment group. The changes in immune responses at the end of cycle 1 (single lenalidomide or single gemcitabine) and cycle 2 (lenalidomide combined with gemcitabine) will be described in relation to baseline within the individual patient. The frequency, magnitude, and quality (Th_1 vs Th_2) of the immune responses will be assessed by the three primary measurements in each patient.

10.2.3 Secondary endpoint (phase II)

- Frequency, intensity and duration of AE and SAE will be subject to descriptive analysis
- Progression-free survival (PFS) will be presented as time from start of therapy until clinical and/or radiological signs of progression of the disease by treatment group together.
- Survival rate at 12 months will be presented as rate of patients still alive at 12 months after start of therapy by treatment group together.
- Overall survival (OS) will be presented as time from start of therapy until death by treatment group together.

10.3 Statistical Methodology

As this is a phase I/II study, only descriptive statistics will be conducted.

10.4 Safety evaluation

Data from all subjects who receive any study drug will be included in the safety analyses. Frequency, intensity and duration of AE and SAE will be subject to descriptive analysis. Subjects who entered the study and did not take any of the study drugs and had this confirmed, will not be evaluated for safety.

The severity of the toxicities will be graded according to the NCI CTCAE v3.0 whenever possible.

10.5 Sample size

This is an explorative phase I/II trial. Nine up to 12 patients will recruited in the phase I part of the trial and additionally 18 up to 21 patients in the phase II.

11 Regulatory Considerations

11.1 Ethics Committee approval

The protocol for this study has been designed in accordance with the general ethical principles outlined in the Declaration of Helsinki [19], Appendix 7. The review of this protocol by the EC and the performance of all aspects of the study, including the methods used for obtaining informed consent, must also be in accordance with principles enunciated in the declaration, as well as ICH Guidelines.

The Investigator will be responsible for preparing documents for submission to the relevant EC and obtaining written approval for this study. The approval will be obtained prior to the initiation of the study.

The approval for both the protocol and informed consent must specify the date of approval, protocol number and version, or amendment number.

Any amendments to the protocol after receipt of EC approval must be submitted by the Investigator to the EC for approval. The Investigator is also responsible for notifying the EC of any serious deviations from the protocol, or anything else that may involve added risk to subjects.

Any advertisements used to recruit subjects for the study must be reviewed and approved by the EC prior to use.

11.2 Informed consent

The Investigator must obtain informed consent, see Appendix 5 and Appendix 6, of a subject or his/her designee prior to any study related procedures as per GCPs and ICH guidelines.

Documentation that informed consent occurred prior to the subject's entry into the study and the informed consent process should be recorded in the subject's source documents. The original consent form signed and dated by the subject and by the person consenting the subject prior to the subject's entry into the study, must be maintained in the Investigator's study files.

11.3 Subject confidentiality

Investigators affirm the subject's right to protection against invasion of privacy, in compliance with local regulations, The Investigator have to permit, when necessary, representatives of regulatory authorities to review and/or copy any medical records relevant to the study in accordance with local laws.

Should direct access to medical records require a waiver or authorization separate from the subject's statement of informed consent, it is the responsibility of the Investigator to obtain such permission in writing from the appropriate individual.

11.4 Study records requirements

The Investigator must ensure that the records and documents pertaining to the conduct of the study and the distribution of the study drug, that is copies of CRFs and source documents (original documents, data, and records [e.g., hospital records; clinical and office charts; laboratory notes; memoranda; subject's diaries or evaluation checklists; pharmacy dispensing records; recorded data from automated instruments; copies or transcriptions certified after verification as being accurate copies; microfiches; photographic negatives, microfilm, or magnetic media; x-rays; subject files; and records kept at the pharmacy, at the laboratories, and at medico-technical departments involved in the clinical study; documents regarding subject treatment and study drug accountability; original signed informed consents, etc.]) be retained by the Investigator for as long as needed to comply with national and international regulations (generally 10 years after discontinuing clinical development or after the last marketing approval). The Investigator agrees to adhere to the document/records retention procedures by signing the protocol.

11.5 Premature discontinuation of study

Celgene reserves the right to terminate this clinical study at any time for reasonable medical or administrative reasons.

Any possible premature discontinuation would be documented adequately with reasons being stated, and information would have to be issued according to local requirements (e.g., EC, Regulatory Authority, etc.).

12 References

1. Alexakis N, Halloran C, Raraty M, Ghaneh P, Sutton R, Neoptolemos JP.Current standards of surgery for pancreatic cancer. Br J Surg. 2004 Nov;91(11):1410-27.

2. Burris HA 3rd et al. Improvements in survival and clinical benefit with gemcitabine as first-line therapy for patients with advanced pancreas cancer: a randomized trial. J Clin Oncol. 1997 Jun;15(6):2403-13.

3. Heinemann V. Gemcitabine in the treatment of advanced pancreatic cancer: a comparative analysis of randomized trials. Semin Oncol (2002) 29(Suppl 20):9–16

4. Hochster HS, Haller DG, de Gramont A, et al. Consensus report of the International Society of Gastrointestinal Oncology on therapeutic progress in advanced pancreatic cancer. Cancer (2006) 107:676–685.

5. Cunningham D, Chau I, Stocken D, et al. Phase III randomised comparison of gemcitabine (GEM) versus gemcitabine plus capecitabine (GEM-CAP) in patients with advanced pancreatic cancer. Eur J Cancer (2005) 3(Suppl):4.

6. Kindler HL et al. Phase II trial of bevacizumab plus gemcitabine in patients with advanced pancreatic cancer. J Clin Oncol. 2005 Nov 1;23(31):8033-40.

7. M.J. Moore, D. Goldstein, J. Hamm, A. Figer, J.R. Hecht and S. Gallinger et al., Erlotinib plus gemcitabine compared with gemcitabine alone in patients with advanced pancreatic cancer: a phase III trial of the National Cancer Institute of Canada Clinical Trials Group, J Clin Oncol 25 (2007), pp. 1960–1966

8. J.J. Densmore, J.R. Fox, G. Kannarkat, J.K. Morgan, G. Petroni and T. Blount et al., A phase I/II trial of weekly gemcitabine with celecoxib and thalidomide for patients with advanced pancreatic cancer, J Clin Oncol 23 (Suppl.) (2005), p. 4241.

9. Dredge K, Horsfall R, Robinson S, Zhang L-H, Lu L, et al. Orally administered lenalidomide (lenalidomide) is anti-angiogenic in vivo and inhibits endothelial cell migration and Akt phosphorylation in vitro. Microvascular Research 69 (2005) 56-63.

10. Corral LF, Haslett PAJ, Muller FW, Chen R, Wong LM, Ocampo CJ, Patterson RT, Stirling DI, Kaplan G. Differential cytokine modulation and T cell activation by two distinct classes of thalidomide analogues that are potent inhibitors of TNF-alpha. J Immunol. 1993;163:380-386.

11. Davies FE, Raje N, Hideshima T, Lentzsch S, Young G, Tai YT, Lin B, Podar K, Gupta D, Chauhan D, Treon SP, Richarson PG, Schlossman RL, Morgan GJ, Muller GW, Stirling DI, Anderson KC. Thalidomide and immunomodulatory derivatives augment natural killer cell cytotoxicity in multiple myeloma. Blood. 2001;98:210-216.

12. Correale P et al: Chemoimmunotherapy of metastatic colorectal.. carcinoma J Clin Oncol Aug 1 ,2005.

13. Bergmann-Leitner ES, Abrams SI. Treatment of human colon carcinoma cell lines with anti-neoplastic agents enhances their lytic sensitivity to antigen-specific CD8+ cytotoxic T lymphocytes. Cancer Immunol Immunother. 2001 Nov;50(9):445-55.

14. Suzuki E, Kapoor V, Jassar AS, Kaiser LR, Albelda SM. Gemcitabine selectively eliminates splenic Gr-1+/CD11b+ myeloid suppressor cells in tumor-bearing animals and enhances antitumor immune activity. Clin Cancer Res. 2005 Sep 15;11(18):6713-21.

15. Amato RJ, Hernandez-McClain J, Saxena S, Khan M. Lenalidomide therapy for metastatic renal cell carcinoma. Am J Clin Oncol. 2008 Jun;31(3):244-9.

16. Sharma RA, Steward WP, Daines CA, Knight RD, O'Byrne KJ, Dalgleish AG. Toxicity profile of the immunomodulatory thalidomide analogue, lenalidomide: phase I clinical trial of three dosing schedules in patients with solid malignancies. Eur J Cancer. 2006 Sep;42(14):2318-25. Epub 2006 Aug 8.

17. Miller AA, Case D, Harmon M, Savage P, Lesser G, Hurd D, Melin SA. Phase I study of lenalidomide in solid tumors. J Thorac Oncol. 2007 May;2(5):445-9

18. Sanborn SL, CooneyM, Gibbons J, Brell J, Savvides P, Krishnamurthi S, Bokar J, Horvath N, Ness A, Remick S. Phase I trial of daily lenalidomide and docetaxel given every three weeks in patients with advanced solid tumours [abstract]. Journal of Clinical Oncology: ASCO Annual Proceedings 2007; 25(18S, Part I of II): 155s, Abstract #3570.

19. World Medical Association. World Medical Association Declaration of Helsinki. Ethical principles for medical research involving human subjects. Nurs Ethics. 2002 Jan;9(1):105-9.

20. Investigators Brochure Revlimid®

21. SmPc Gemzar®

22. Crane E, List A. Immunomodulatory drugs. Cancer Investigations 2005;23(7):625-34.

23. Bartlett JB, Michael A, Clarke IA, Dredge K, Nicholson S, Kristeleit H et al. Phase I study to determine the safety, tolerability and immunostimulatory activity of thalidomide analogue CC-5013 in patients with metastatic malignant melanoma and other advanced cancers. Br J Cancer. 2004 Mar 8;90(5):955-61.

24. Liu WM, Nizar S, Dalgleish AG. Gemcitabine and lenalidomide combination in a patient with metastatic pancreatic cancer: a case study. Published Online 12 May 2009.

Appendices

Appendix 1 – ECOG Performance Status Scale

SCORE	DESCRIPTION
0	Fully active, able to carry on all pre-disease performance without restriction.
1	Restricted in physically strenuous activity but ambulatory and able to carry out work of a light or sedentary nature, e.g., light house work, office work.
2	Ambulatory and capable of all self-care but unable to carry out any work activities. Up and about more than 50% of waking hours.
3	Capable of only limited self-care, confined to bed or chair more than 50% of waking hours.
4	Completely disabled. Cannot carry on any self-care. Totally confined to bed or chair.
5	Dead.

Appendix 4 – SAE Form

Karolinska Universitetssjukhuset Solna

Page 1(2)

Serious Adverse Event SAE Form

LENAGEM-PANC study for Pancreas cancer patients

☐ Initial Report	☐ Follow up Report #
Patient Nr:	
Date:	
Principal Investigator: Maria Liljefors<br>Sponsor: Håkan Mellstedt	

Patient demographics

Patient initials	Sex	Date of birth DD-MM-YYYY	Height	Weight
	☐ M ☐ F		cm	kg

Study Medication

Study drug name: Start date DD-MM-YYYY	Stop date DD-MM-YYYY	Dose (route, unit /time interval)
Batch Nr:<br>Drug code broken<br>☐ Yes ☐ No ☐ NA	☐ Continues	

Serious Adverse Event

Event in medical terms	Onset date DD-MM-YYYY	Stop date DD-MM-YYYY	Severity
	Time of onset (if known)	☐ Continues	Mild ☐<br>Moderate ☐<br>Severe ☐

Serious Criteria	Assessment of Causality	Event Outcome
Death date: __________<br>Autopsy: ☐ Yes ☐ No<br>Life threatening ☐<br>Inpatient or prolonged hospitalisation ☐<br>Persistent or significant disability/incapacity ☐<br>Congenital Anomaly or Birth Defect ☐	☐ Not suspected<br>☐ Suspected*<br><br>* If suspected, report to MPA all cases that involves death, life threatening reaction or any unexpected serious adverse reaction (SUSAR)	☐ Resolved Completely<br>date: __________<br>☐ Resolved with Sequel<br>☐ Resolving<br>☐ Unresolved<br>☐ Death<br>☐ Unknown

Action taken

☐ No action taken ☐ Trial drug dosage adjusted ☐ Trial drug permanently adjusted ☐ Non-drug therapy given

☐ Concomitant medication adjusted

PATIENTENS INFORMERADE SAMTYCKE

"LenaGem" – EN FAS I STUDIE AVSEENDE BEHANDLING MED REVLIMID ® (LENALIDOMID) och GEMZAR® (GEMCITABIN) HOS PATIENTER MED CANCER I BUKSPOTTKÖRTELN

Inledning

Du tillfrågas härmed om du vill delta i en forskningsstudie för patienter med cancer i bukspottkörteln. Som du vet så är din sjukdom svår att behandla framgångsrikt, varför du tillfrågas om du vill var med i den nu aktuella forskningsstudien. Innan du beslutar dig för om du vill delta eller inte, är det viktigt att du förstår varför studien genomförs och vad den kommer att innebära för dig om du samtycker till att delta. Läs följande information noggrant. Ställ gärna frågor om det finns något som du inte förstår eller om du vill ha mer information. Du får gärna diskutera med dina närstående innan du bestämmer dig. Ditt deltagande i denna studie är frivilligt, och du kan när som helst och utan att behöva ange orsaken avbryta ditt deltagande i studien. Om du tackar nej så kommer det inte att påverka ditt framtida omhändertagande.

Bakgrund och syfte med denna studie

Gemcitabin (Gemzar®) är en cytostatikabehandling, som är registrerad för behandling mot cancer i bukspottkörteln. Detta är också den vedertagna standardbehandlingen. Gemzar® är effektivt hos vissa men inte merparten av patienterna, varför det är angeläget att försöka finna än mer effektiva behandlingssätt. Ett sätt kan vara att kombinera Gemzar® med andra verksamma mediciner, sådana som inte består av cellgift utan verkar på andra sätt.

Lenalidomid (Revlimid®) är ett läkemedel som tillhör en grupp av läkemedel som har tumör-hämmande effekt, delvis genom att hämma blodkärlsnybildningen (s.k. anti-angiogena egenskaper) i tumören men också genom att reglera funktioner i immunsystemet. Det har redan godkänts i Europa och USA för användning vid en blodsjukdom (myelom). Revlimid® är en modifierad motsvarighet till läkemedlet thalidomid (Neurosedyn), som man vet är fosterskadande hos människa, troligtvis via dess anti-angiogena egenskaper och orsakar allvarliga, livshotande, medfödda missbildningar. Detta är även en risk med Revlimid®, och om läkemedlet tas under graviditet, kan det orsaka medfödda missbildningar eller död hos ett ofött barn. Detta har man också sett i studier på apor.

Om du är kvinna i fertil ålder måste du samtycka till att inte bli gravid under tiden du tar Revlimid®. Graviditetstest måste tas före studien, därefter var fjärde vecka under studien pågår samt fyra veckor efter avslutad behandling. Du måste också använda effektivt preventivmedel under hela studien. Om du är man med sexuell kontakt med kvinna i fertil ålder måste du använda kondom vid samlag, eftersom lenalidomid har kunnat påvisas i sädesvätska.

Du får aldrig ge Revlimid® (eller andra studieläkemedel) till någon annan. Du får aldrig donera blod under tiden du deltar i denna studie och under minst en månad efter att du har slutat med behandlingen.

Denna studie är en s.k. Fas I studie där Revlimid® kommer att användas i tre olika dosnivåer. De tre första inkluderade patienterna kommer att erhålla Revlimid® i dos 15 mg en gång dagligen i 21 dagar. Om det inte uppkommit några oacceptabla biverkningar hos dessa tre patienter kommer dosen att höjas till 20 mg Revlimid® dagligen i 21 dagar till ytterligare tre patienter. De sista tre inkluderade patienterna kommer att erhålla Revlimid® i dos 25 mg en gång dagligen i 21 dagar om inga oacceptabla biverkningar uppkommit på de lägre dosnivåerna.

Syftet med denna studie är att bedöma vilken dosnivå av Revlimid® som är säker och acceptabel ur biverkningssynpunkt när Revlimid® kombineras med Gemzar® vid behandling av patienter med cancer i bukspottkörteln hos patienter som tidigare inte erhållit någon cellgiftsbehandling för tumörsjukdomen. Den dos av Revlimid® som bedöms vara lämplig att använda i kombination med Gemzar®, kommer därefter att användas i en fortsättningsdel av studien.

I denna studie kommer totalt 9 patienter att ingå. Om det anses angeläget att inkludera ytterligare patienter, kan ytterligare 1, upp till 3 patienter till komma att delta.

Vad omfattar studien?

Studien består av följande olika delar: en undersökningsfas och behandlingsfas.

Undersökningsfas: Om du bestämmer dig för att vilja delta i studien, måste vi först ta reda på om du kan vara med. Undersökningsproceduren omfattar: sjukdomshistoria, läkemedel som du tar, vanlig kroppsundersökning, röntgenundersökningar, EKG, och blodprover. Detta är undersökningar som väsentligen skulle göras även om du ej skulle delta i studien. Eftersom Revlimid® kan ha fosterskadande effekter, måste man också göra en genomgång av preventivmetod, och graviditetstest behöver utföras innan behandlingen börjar om du är kvinna i barnafödande ålder.

Behandlingsfas: Om förtesterna visar att du kan vara med i studien, kommer du att starta behandling med Revlimid®-kapslar i 21 dagar, och därefter uppehåll i 7 dagar. Du får också Gemzar® som intravenös infusion (dropp) under 30 min, dag 1, 8 och 15. Detta utgör den första behandlingscykeln (cykel 1). Läkemedelsbehandling kommer sedan att fortsätta tills dess att du inte tål den eller att den är overksam.

Under varje behandlingscykel kommer du att stå under noggrann kontroll och tillsyn. Blodprover kommer att tas ca var 4e dag under de första två behandlingsveckorna och dag 21, 22 eller 23 under den första behandlingscykeln. Från och med den andra behandlingscykel och framåt, tas blodprover innan start av behandlingscykeln, därefter en gång i veckan fram till dag 21, 22 eller dag 23 i cykeln. Från och med cykel tre och framåt tas blodprover innan start av behandling, därefter en gång i veckan under de första två veckorna. Jämfört med standardbehandling med enbart Gemzar®, tas fyra

extra blodprov för att kontrollera nivån av vita blodkroppar och blodplättar under behandlingen.

Om du upplever några besvär eller obehag under behandlingen är det viktigt att du direkt rapporterar detta.

Om du drabbas av biverkningar, kan läkaren göra en paus i behandlingen, ge en lägre dos av medicinerna, eller avsluta behandlingen beroende på grad och typ av biverkning och hur snabbt den går tillbaka.

Under behandlingen är det läkarbesök 1 gång i månaden, dvs. i samband med start av varje ny behandlingscykel. Vid dessa besök kommer vi att gå igenom dina läkemedel, ditt medicinska tillstånd, göra vanlig kroppsundersökning, ta blodprover och mäta puls, blodtryck och vikt. Totalt kommer det under den första behandlingscykeln tas ca 10 ml extra blod/vecka, dvs. 1 extra provrör/vecka, under den andra cykeln tas ca 10 ml extra blod, dvs. 1 extra provrör utöver rutin. Från och med den tredje cykeln och framåt tas blodprover enligt rutin dvs en gång per vecka under tre veckor.

Vad finns det för risker med studien?

Det innebär alltid en risk att ta läkemedel, men du kommer att bli noggrant övervakad med avseende på eventuella biverkningar. Du ska inte tveka att rapportera allt som oroar dig eller besvärar dig till din läkare, även om du tror att det inte har samband med att du tar studieläkemedlen. Om du har några frågor ska du kontakta läkaren eller forsknings-sköterskan.

Övriga läkemedel som kommer att ingå i behandlingen:

Då det finns en viss ökad risk för blodproppsbildning (djup ventrombos eller blod-proppsbildning i lungorna) till följd av din underliggande tumörsjukdom, cytostatikabehandlingen och vid behandling med lenalidomid, kommer du att erhålla behandling med blodproppsförebyggande medicin i förebyggande dos. Dessa läkemedel ges lämpligen i form av en spruta för daglig injektion i huden och vi kommer att diskutera igenom detta med dig. Du kommer att få instruktioner om hur denna spruta ges i syfte att du kan ta den själv eller få hjälp av någon anhörig eller vårdpersonal. Du behöver således inte vara på sjukhuset för dessa injektioner.

Möjliga biverkningar av Revlimid®:

Revlimid® har undersökts hos friska frivilliga försökspersoner och hos patienter med olika typer av cancer. Liksom när det gäller annan försöksbehandling kan det finnas biverkningar eller risker förknippade med Revlimid®, och det kan finnas sådana som ännu inte är kända. Nedan anges de biverkningar som har rapporterats hos 5 500 patienter, som har deltagit i tidigare och i pågående cancerstudier med Revlimid®. Dessa händelser ansågs av studieläkarna kunna ha möjligt samband med Revlimid®. Vi är medvetna om att listan nedan är lång och kan vara svår att läsa och tolka, varför du inte ska tveka att be din läkare att förklara.

*Vanliga biverkningar (*rapporterades *hos mer än 20% av patienterna):*

är medvetna om att listan nedan är lång och kan vara svår att läsa och tolka, varför du inte ska tveka att be din läkare att förklara.

Vanliga biverkningar (rapporterades hos mer än 20% av patienterna):
Neutropeni (minskat antal vita blodkroppar). Trombocytopeni (minskat antal blodplättar).

Mindre vanliga biverkningar (rapporterades hos mellan 3 och 20% av patienterna):
Hudutslag; klåda och torr hud; diarré; förtoppning; illamående; kräkningar; smakförändringar; aptitförlust; muntorrhet; muskelkramper; muskelsmärtor; ömma leder; avsaknad eller förlust av styrka; anemi (blodbrist); feber; yrsel; sömnsvårigheter; svullnad i armar och ben; huvudvärk; skakningar; smärta; stickningar; förändrad känsel i händer eller fötter; andfåddhet.

Sällsynta men i vissa fall allvarliga biverkningar (mindre än 3% av patienterna)
Följande biverkningar har rapporterats hos enstaka patienter som fått Revlimid®:

Djup ventrombos eller blodpropp i lungorna. Om denna biverkan uppstår kommer du att behandlas med blodförtunnande läkemedel i högre dos än den du får som blodkroppsförebyggande. Blödningsbenägenhet; hemolys (ökad nedbrytning av röda blodkroppar); bakterie- och virusinfektioner; ökad eller minskad hjärtfrekvens; onormala eller oregelbundna hjärtslag; nedsatt förmåga hos hjärtat att effektivt pumpa blod; sänkt blodtryck; hjärtstillestånd; depression; svimning; stroke; minskat blodflöde till hjärnan; kramper; lever eller njursvikt; förhöjda blodsockernivåer; sköldkörtelpåverkan; uttorkning; allergisk reaktion; ökat tryck i lungornas blodkärl.

Följande möjliga biverkningar rapporterades vardera hos en patient:

Buksmärta; inflammation i mun- och halsslemhinnor; minskat blodflöde till tarmarna; inflammation i tarmarna; binjuresvikt; vätske- och elektrolytrubbningar; förhöjd bilirubinnivå i blodet; blod i urinen; oförmåga att urinera; bröstsmärta; förhöjt blodtryck; sänkt blodtryck; ledbesvär; aptitförlust; upphostning av blod; försämrade astmasymtom; inflammation och fibros i lungorna, andningssvårigheter, minnesförsämring, vanföreställningar; dimsyn; ansiktssvullnad; basalcellscancer och hudlymfom; hjärntumör; och multipelt myelom (en annan typ av blodcancer).

Skulle ytterligare viktig säkerhetsinformation kring Revlimid® framkomma så kommer du att omgående att få reda på detta.

Möjliga biverkningar av Gemzar®:
Behandling med Gemzar® kan bl.a. ge illamående, kräkningar, reducerat antal blodkroppar, hudreaktioner, diarré, feber, huvudvärk, influensaliknande symtom, större infektionsrisk, inflammation vid infusionsstället och bröstsmärta.

Båda läkemedlen kan ge en övergående sänkning av de vita blodkropparna, granulocyterna, och i studien kan man ge de patienter som behöver en granulocytstimulerande medicin. Detta ges som sprutor under huden, och dosen justeras

individuellt så att granulocyterna i blodet ligger på rätt nivå. Likaså kan båda läkemedlen ge en övergående minskning av blodplättarna (trombocyterna), vilket kan öka risken för blödningar från slemhinnor i mun, magtarmkanal etc. Det kan då bli aktuellt att tillfälligt sätta ut det blodproppförebyggande läkemedlet som Du tar som daglig injektion i huden tills trombocytvärdet stigit igen.

Har jag några fördelar av att delta i studien?

Det finns inga garantier för att en bättre effekt kan uppnås genom att kombinera läkemedlen såsom i denna studie, men med tanke på deras respektive verkningsmekanismer kan det vara möjligt.

Vilka andra alternativ finns det?

Om du väljer att inte vara med i studien, så kommer du erbjudas behandling enl normal standardrutin på din klinik. Den vanligaste behandlingen i detta läge är att ge Gemzar® enbart, d.v.s. utan Revlimid®.

Sekretess och användande av studieresultaten

Den information som samlats in under studiedeltagandet ställs samman och behandlas av forskarna i syfte att få kunskap om behandlingens effekter och eventuella biverkningar. Resultaten kommer också att monitoreras av oberoende person (från annan klinik) med tystnadsplikt. Den kodade informationen kan komma att publiceras i medicinsk vetenskaplig tidskrift och kan komma att lämnas till berörd myndighet. Endast den ansvarige läkaren kan med hjälp av koden identifiera dig som person. Studien kommer att rapporteras som en sammanställning av grupper av patienter. Dina individuella data kommer inte att vara möjliga att identifiera.

Forskningsprover i biobank

Prover som tas i samband med denna forskningsstudie kommer att förvaras i en så kallad biobank. Detta sker uteslutande vid vårt forskningslaboratorium vid Karolinska Universitetssjukhuset och inga prover kommer att lämna sjukhuset. De immunologiska testernas resultat kommer att finnas tillgängliga i slutet av studien. Proverna kommer att förvaras kodade, vilket innebär att de inte kan spåras direkt till dig som person. Proverna och en identifieringslista (kodlista) kommer att förvaras på ett säkert ställe och hållas åtskilda.

Du har inflytande över de prover som lagras på detta sätt genom att de kommer att förstöras om du så begär. Genom att ge ditt samtycke till att delta i studien samtycker du också till att vi förvarar prover i biobanken enl. ovan.

Proverna kommer endast att användas på det sätt som du givit samtycke till i studien och kan endast användas för ev nya forskningsprojekt efter att du har lämnat nytt samtycke och/eller godkännande har erhållits från den etiska granskningsnämnden.

Försäkring och ersättning

Kompensation för skada hos patient eller frivilliga försökspersoner, vilken uppstår i samband med medicinsk forskning betalas i enlighet med bestämmelserna i patientskadelagen (SFS 1996:799). Du kommer inte att få betalt för att du deltar i denna studie. Revlimid® tillhandahålles utan kostnad under tiden studien pågår.

Frivillighet
Deltagandet i denna studie är helt frivilligt. Du får avbryta ditt deltagande i studien när som helst utan att behöva uppge något skäl. Ett beslut att avbryta deltagandet eller att inte delta i denna studie kommer inte att påverka kvaliteten på den vård som du får. Ditt deltagande kan avbrytas om din läkare eller studieledningen anser att det är bäst för dig, eller om Läkemedelsverket beslutar att avbryta studien. Du kommer att informeras om ev viktiga nya fynd som framkommer under studiens gång, vilka kan påverka ditt fortsatt deltagande.

Studiekontakter
Om du har frågor om dina rättigheter som studiedeltagare, eller vill få mer information – ta då kontakt med någon av de ansvariga personer som anges nedan eller med din egen läkare eller forskningssköterskan.

Maria Liljefors, överläkare på onkologkliniken.
Radiumhemmet, Karolinska universitetssjukhuset, Solna, 171 76 Stockholm.
Telefon: 08-51776136

INFORMERAT SAMTYCKE

Jag har fått muntlig och skriftlig information om studien avseende Revlimid® i kombination med Gemzar® till patienter med cancer i bukspottkörteln. Jag har haft möjlighet att ställa frågor och mina frågor har blivit besvarade. Jag samtycker till att delta i denna studie och gör det av egen fri vilja. Jag förstår att jag när som helst och utan förklaring kan avsluta mitt deltagande i studien utan att detta påverkar min framtida behandling och vård.

Jag samtycker också till att mina prover förvaras i en s.k. biobank och att resultaten får granskas (monitoreras) av annan person (med tystnadsplikt) än de för studien direkt ansvariga.

Jag har informerats och samtycker till att personal från svensk läkemedelsmyndighet jämför de data som rapporteras i denna studie med vad som finns i min patientjournal. Jag ger mitt samtycke, under förutsättning att den information som därmed blir tillgänglig inte förs vidare.

Jag har fått en kopia av Patientinformationen.

....

PATIENTENS NAMNTECKNING **DATUM**

....

TEXTAT NAMN

Jag intygar att jag har informerat patienten muntligen och skriftligen om syftet med denna studie och vad ett deltagande kan innebära.

....

LÄKARENS NAMNTECKNING **DATUM**

....

TEXTAT NAMN

TILLÄGG TILL PATIENTENS INFORMERADE SAMTYCKE

Jag accepterar att proverna används i framtida forskning som inte är beskriven här och som i förekommande fall kommer att granskas och godkännas av regional etikprövningsnämnd (Stryks om patienten motsätter sig det).

JA____________ NEJ______________

....

PATIENTENS NAMNTECKNING

Appendix 7

WORLD MEDICAL ASSOCIATION DECLARATION OF HELSINKI
Ethical Principles for Medical Research Involving Human Subjects

Adopted by the 18th WMA General Assembly, Helsinki, Finland, June 1964, and amended by the:
29th WMA General Assembly, Tokyo, Japan, October 1975
35th WMA General Assembly, Venice, Italy, October 1983
41st WMA General Assembly, Hong Kong, September 1989
48th WMA General Assembly, Somerset West, Republic of South Africa, October 1996
52nd WMA General Assembly, Edinburgh, Scotland, October 2000
53th WMA General Assembly, Washington 2002 (Note of Clarification on paragraph 29 added)
55th WMA General Assembly, Tokyo 2004 (Note of Clarification on Paragraph 30 added)
59th WMA General Assembly, Seoul, October 2008

A. INTRODUCTION

1. The World Medical Association (WMA) has developed the Declaration of Helsinki as a statement of ethical principles for medical research involving human subjects, including research on identifiable human material and data.

   The Declaration is intended to be read as a whole and each of its constituent paragraphs should not be applied without consideration of all other relevant paragraphs.

2. Although the Declaration is addressed primarily to physicians, the WMA encourages other participants in medical research involving human subjects to adopt these principles.

3. It is the duty of the physician to promote and safeguard the health of patients, including those who are involved in medical research. The physician's knowledge and conscience are dedicated to the fulfilment of this duty.

4. The Declaration of Geneva of the WMA binds the physician with the words, "The health of my patient will be my first consideration," and the International Code of Medical Ethics declares that, "A physician shall act in the patient's best interest when providing medical care."

5. Medical progress is based on research that ultimately must include studies involving human subjects. Populations that are underrepresented in medical research should be provided appropriate access to participation in research.

6. In medical research involving human subjects, the well-being of the individual research subject must take precedence over all other interests.

7. The primary purpose of medical research involving human subjects is to understand the causes, development and effects of diseases and improve preventive, diagnostic and therapeutic interventions (methods, procedures and treatments). Even the best current interventions must be evaluated continually through research for their safety, effectiveness, efficiency, accessibility and quality.

8. In medical practice and in medical research, most interventions involve risks and burdens

9. Medical research is subject to ethical standards that promote respect for all human subjects and protect their health and rights. Some research populations are particularly vulnerable and need special protection. These include those who cannot give or refuse consent for themselves and those who may be vulnerable to coercion or undue influence.

10. Physicians should consider the ethical, legal and regulatory norms and standards for research involving human subjects in their own countries as well as applicable international norms and standards. No national or international ethical, legal or regulatory requirement should reduce or eliminate any of the protections for research subjects set forth in this Declaration.

B. BASIC PRINCIPLES FOR ALL MEDICAL RESEARCH

11. It is the duty of physicians who participate in medical research to protect the life, health, dignity, integrity, right to self-determination, privacy, and confidentiality of personal information of research subjects..

12. Medical research involving human subjects must conform to generally accepted scientific principles, be based on a thorough knowledge of the scientific literature, other relevant sources of information, and adequate laboratory and, as appropriate, animal experimentation. The welfare of animals used for research must be respected.

13. Appropriate caution must be exercised in the conduct of medical research that may harm the environment.

14. The design and performance of each research study involving human subjects must be clearly described in a research protocol. The protocol should contain a statement of the ethical considerations involved and should indicate how the principles in this Declaration have been addressed. The protocol should include information regarding funding, sponsors, institutional affiliations, other potential conflicts of interest, incentives for subjects and provisions for treating and/or compensating subjects who are harmed as a consequence of participation in the research study. The protocol should describe arrangements for post-study access by study subjects to interventions identified as beneficial in the study or access to other appropriate care or benefits.

15. The research protocol must be submitted for consideration, comment, guidance and approval to a research ethics committee before the study begins. This committee must be independent of the researcher, the sponsor and any other undue influence. It must take into consideration the laws and regulations of the country or countries in which the research is to be performed as well as applicable international norms and standards but these must not be allowed to reduce or eliminate any of the protections for research subjects set forth in this Declaration. The committee must have the right to monitor ongoing studies. The researcher must provide monitoring information to the committee, especially information about any serious adverse events. No change to the protocol may be made without consideration and approval by the committee.

16. Medical research involving human subjects must be conducted only by individuals with the appropriate scientific training and qualifications. Research on patients or healthy volunteers requires the supervision of a competent and appropriately qualified physician

25. For medical research using identifiable human material or data, physicians must normally seek consent for the collection, analysis, storage and/or reuse. There may be situations where consent would be impossible or impractical to obtain for such research or would pose a threat to the validity of the research. In such situations the research may be done only after consideration and approval of a research ethics committee.

26. When seeking informed consent for participation in a research study the physician should be particularly cautious if the potential subject is in a dependent relationship with the physician or may consent under duress. In such situations the informed consent should be sought by an appropriately qualified individual who is completely independent of this relationship.

27. For a potential research subject who is incompetent, the physician must seek informed consent from the legally authorized representative. These individuals must not be included in a research study that has no likelihood of benefit for them unless it is intended to promote the health of the population represented by the potential subject, the research cannot instead be performed with competent persons, and the research entails only minimal risk and minimal burden.

28. When a potential research subject who is deemed incompetent is able to give assent to decisions about participation in research, the physician must seek that assent in addition to the consent of the legally authorized representative. The potential subject's dissent should be respected.

29. Research involving subjects who are physically or mentally incapable of giving consent, for example, unconscious patients, may be done only if the physical or mental condition that prevents giving informed consent is a necessary characteristic of the research population. In such circumstances the physician should seek informed consent from the legally authorized representative. If no such representative is available and if the research cannot be delayed, the study may proceed without informed consent provided that the specific reasons for involving subjects with a condition that renders them unable to give informed consent have been stated in the research protocol and the study has been approved by a research ethics committee. Consent to remain in the research should be obtained as soon as possible from the subject or a legally authorized representative.

30. Authors, editors and publishers all have ethical obligations with regard to the publication of the results of research. Authors have a duty to make publicly available the results of their research on human subjects and are accountable for the completeness and accuracy of their reports. They should adhere to accepted guidelines for ethical reporting. Negative and inconclusive as well as positive results should be published or otherwise made publicly available. Sources of funding, institutional affiliations and conflicts of interest should be declared in the publication. Reports of research not in accordance with the principles of this Declaration should not be accepted for publication.

C. ADDITIONAL PRINCIPLES FOR MEDICAL RESEARCH COMBINED WITH MEDICAL CARE

31. The physician may combine medical research with medical care only to the extent that the research is justified by its potential preventive, diagnostic or therapeutic value and if the physician has good reason to believe that participation in the research study will not adversely affect the health of the patients who serve as research subjects.

32. The benefits, risks, burdens and effectiveness of a new intervention must be tested against those of the best current proven intervention, except in the following circumstances:
    - The use of placebo, or no treatment, is acceptable in studies where no current proven intervention exists; or
    - Where for compelling and scientifically sound methodological reasons the use of placebo is necessary to determine the efficacy or safety of an intervention and the patients who receive placebo or no treatment will not be subject to any risk of serious or irreversible harm. Extreme care must be taken to avoid abuse of this option.

33. At the conclusion of the study, patients entered into the study are entitled to be informed about the outcome of the study and to share any benefits that result from it, for example, access to interventions identified as beneficial in the study or to other appropriate care or benefits.

34. The physician must fully inform the patient which aspects of the care are related to the research. The refusal of a patient to participate in a study or the patient's decision to withdraw from the study must never interfere with the patient-physician relationship.

35. In the treatment of a patient, where proven interventions do not exist or have been ineffective, the physician, after seeking expert advice, with informed consent from the patient or a legally authorized representative, may use an unproven intervention if in the physician's judgement it offers hope of saving life, re-establishing health or alleviating suffering. Where possible, this intervention should be made the object of research, designed to evaluate its safety and efficacy. In all cases, new information should be recorded and, where appropriate, made publicly available.

Patientdagbok för studiemedicinering Dag 1-21

Dos av studieläkemedlet *lenalidomid*:_______________mg (per dag)

Pat nr:_____________ Pat initialer:______________ Cykel: #_____________

Fyll i varje dag tablett av den styrka som du tar. Om du missat någon dos, skriv anledningen

Tänk på att:

- ta tabletten vid samma tidpunkt varje dag
- tabletten får inte krossas eller tuggas
- tabletten ska sväljas hel, helst med vatten, med eller utan mat
- om det gått mindre än 12 timmar sedan du egentligen skull ta din tablett, så kan du ta den
- om det gått mer än 12 timmar sedan du egentligen skulle ha tagit din tablett, ska du inte ta dosen utan ta nästa dos vid normal tidpunkt följande dag
- om du kräkts upp din tablett, ta ingen ny utan ta nästkommande dag som vanligt

Dag	Datum	Tid (00:00)	Veckodag	5 mg	10 mg	15 mg	20 mg	25 mg	Anledning till missad dos
1									
2									
3									
4									
5									
6									
7									

Dag	Datum	Tid (00:00)	Veckodag	5 mg	10 mg	15 mg	20 mg	25 mg	Anledning till missad dos
8									
9									
10									
11									
12									
13									
14									
15									
16									
17									
18									
19									
20									
21									
